# Integrative Metabolic Signatures for Hepatic Radiation Injury

**DOI:** 10.1371/journal.pone.0124795

**Published:** 2015-06-05

**Authors:** Irwin Jack Kurland, Pilib Ó. Broin, Aaron Golden, Gang Su, Fan Meng, Laibin Liu, Robert Mohney, Shilpa Kulkarni, Chandan Guha

**Affiliations:** 1 Department of Medicine, Diabetes Center, Stable Isotope and Metabolomics Core Facility, Albert Einstein College of Medicine, Bronx, New York, United States of America; 2 Division of Computational Genetics, Genetics Department, Albert Einstein College of Medicine, Bronx, New York, United States of America; 3 Department of Computational Medicine and Bioinformatics, University of Michigan, Ann Arbor, Michigan, United States of America; 4 Department of Radiation Oncology, Albert Einstein College of Medicine, Bronx, New York, Unites States of America; 5 Metabolon, Durham, North Carolina, United States of America; Korea University, KOREA, REPUBLIC OF

## Abstract

**Background:**

Radiation-induced liver disease (RILD) is a dose-limiting factor in curative radiation therapy (RT) for liver cancers, making early detection of radiation-associated liver injury absolutely essential for medical intervention. A metabolomic approach was used to determine metabolic signatures that could serve as biomarkers for early detection of RILD in mice.

**Methods:**

Anesthetized C57BL/6 mice received 0, 10 or 50 Gy Whole Liver Irradiation (WLI) and were contrasted to mice, which received 10 Gy whole body irradiation (WBI). Liver and plasma samples were collected at 24 hours after irradiation. The samples were processed using Gas Chromatography/Mass Spectrometry and Liquid Chromatography/Mass Spectrometry.

**Results:**

Twenty four hours after WLI, 407 metabolites were detected in liver samples while 347 metabolites were detected in plasma. Plasma metabolites associated with 50 Gy WLI included several amino acids, purine and pyrimidine metabolites, microbial metabolites, and most prominently bradykinin and 3-indoxyl-sulfate. Liver metabolites associated with 50 Gy WLI included pentose phosphate, purine, and pyrimidine metabolites in liver. Plasma biomarkers in common between WLI and WBI were enriched in microbial metabolites such as 3 indoxyl sulfate, indole-3-lactic acid, phenyllactic acid, pipecolic acid, hippuric acid, and markers of DNA damage such as 2-deoxyuridine. Metabolites associated with tryptophan and indoles may reflect radiation-induced gut microbiome effects. Predominant liver biomarkers in common between WBI and WLI were amino acids, sugars, TCA metabolites (fumarate), fatty acids (lineolate, n-hexadecanoic acid) and DNA damage markers (uridine).

**Conclusions:**

We identified a set of metabolomic markers that may prove useful as plasma biomarkers of RILD and WBI. Pathway analysis also suggested that the unique metabolic changes observed after liver irradiation was an integrative response of the intestine, liver and kidney.

## Introduction

Radiation-induced liver disease (RILD) was initially described several decades ago as a potentially lethal delayed effect of conventionally fractionated whole liver radiation therapy (RT) with histopathological features of hepatic central veno-occlusive disease [1, 2]. RT, alone or in combination with chemotherapy, can cure many types of solid tumors such as prostate, head and neck, cervix, and anal cancers. However, poor radiotolerance of liver prohibits RT and chemotherapy from playing a curative role in the treatment of liver cancers. Classic RILD typically manifests in patients with non-cirrhotic liver, 2 to 4 months after completion of whole liver RT, with symptoms of fatigue, increase of abdominal girth, occasional ascites and an increase in alkaline phosphatase without any other abnormalities in liver function tests, including jaundice or increase in serum ammonia levels [3, 4]. The pathophysiology of classic RILD is attributed to radiation-induced injury to the liver sinusoidal endothelial cells, which ultimately induces a sinusoidal obstruction syndrome (SOS) and occlusion of the hepatic central veins. In patients with cirrhotic liver, RT causes a more acute syndrome with an increase in serum transaminases indicating hepatocellular injury, possibly in regenerating nodules. In recent years, with the advent of improved three-dimensional RT treatment planning [5] and image guidance, several investigators have demonstrated that large doses of RT in conventional or hypofractionated regimens can be delivered to partial volumes of liver without causing lethal RILD [6–11]. While these studies have raised hope of cure for patients with small liver cancers, RILD still remains a concern for patients with larger tumors or with cirrhotic livers because definitive doses of RT cannot be delivered in such situations.

One of the limitations of diagnosing RILD is that the clinical signs and symptoms develop late in the disease and there are no early clinical biomarkers available. Furthermore, most of the pathophysiological insight to the mechanisms of RILD was obtained from autopsies of patients with terminal RILD [12, 13]. Although these studies highlight the importance of radiation-induced sinusoidal/endothelial injury in RILD, studies in rodents [14, 15] and non-human primates [16] have demonstrated that radiation adversely modulates the function of parenchymal hepatocytes, such as, inhibition of regeneration, receptor-mediated endocytosis and biliary clearance. The effect of radiation on cellular metabolism has been studied for a number of years to understand the cellular oxidative balance and DNA repair upon exposure to ionizing radiation (IR) [17, 18]. However, the effect of RT in altering the metabolic pathways has not been studied systemically. To date, a comprehensive analysis of the biochemical changes resulting from liver irradiation has not been conducted. In our quest to develop sensitive and early markers of hepatic radiation injury for efficient monitoring of RILD and to better understand the biochemical mechanisms contributing to hepatic radiation toxicity that precedes RILD, metabolomic profiles of liver and plasma following WLI (10, 50 Gy) were generated. Fifty Gy WLI dose was chosen based on the histopathological confirmation of RILD observed in our earlier studies [15]. Ten Gy WLI dose was chosen to determine whether a lower radiation dose that does not results in RILD but can potentially have latent effects could cause detectable metabolic changes. Ten Gy WBI dose, which is lethal in mice, was used to compare the differences between liver exposure and multi-organ exposure response systemically (plasma) and at tissue level (liver). Furthermore, comparisons of plasma metabolite signatures obtained in 10 Gy WLI, 50 WLI and 10 Gy WBI were also conducted to determine the sensitivity of different biochemical pathways to WLI and WBI. Samples were collected 24 hours post irradiation, which provided sufficient window to detect the effects of radiation-induced transcriptional, and translational changes on metabolomic profile.

We used metabolomics approach because it enables detection and quantification of small molecules involved in cellular metabolic networks and associated signaling pathways. Metabolites are the final products of cellular regulatory processes, and their quantitative levels can be regarded not only as the response of biological systems to genetic, environmental and therapy-related changes, but as controllers to preserve fuel homeostasis and tissue specific fuel utilization. Global metabolomic profiling of biological fluids can uncover latent, endogenous small molecules (<850–900 daltons) [19]. Metabolites have a bimodal size distribution, having more than 30% in the 100–400 Da range and a similar number in the 700–900 Da range [20]. Adding lipidomic profiling extends the “net” of biomarker discovery, and lipids may be up to ~1500–2000 daltons.

Currently there is a lack of biomarkers that correlate with radiation injury of various organs. The liver, in particular, could be affected by any protocol involving abdominal radiation. In this study, plasma radiation biomarkers were found that correlated with liver radiation biomarkers, and also suggested significant metabolic interactions between intestine/microbiome, kidney and liver are part of the response to upper abdominal irradiation. Organ-Organ metabolic interactions that could depend on gut microflora should not be surprising, as a number of metabolomics studies have shown that there is significant interplay between bacterial and mammalian metabolism that helps determine the plasma metabolome [21–23]. The insights this study provides regarding metabolic mechanisms enable the understanding of the key compensatory metabolic pathways for RILD, which may point to therapy development.

## Experimental Procedures

### WLI and WBI and experimental cohorts

All animal studies were performed under approved institutional protocols and according to the guidelines established in the Guide for the Care and Use of Laboratory Animals. The animal protocols were in accordance with IACUC (Institutional Animal Care and Use Committee) of the Albert Einstein College of Medicine.

WLI was performed as previously described [15, 24]. Briefly, 8-to-10 week old male C57Bl/6 mice were anesthetized by isofluorane inhalation using a closed circuit. After receiving a midline incision, animals were positioned on a specially constructed polystyrene (aquaplast) platform. A jig was aligned on aquaplast and separated into two compartments through which a longitudinal port (5 x 7 cm) was accessible for irradiation. Two 2-mm thick, 3 x 4 cm lead shields were wedged beneath the liver to displace the stomach and intestines without compressing the hepatic and aortic vessels. A separate lead shield was placed 0.5 cm above the xyphoid process superiorly to limit the lung dose. WLI was delivered through the use of a single anterior beam using a 320 MGC Philips orthovoltage unit operating at 320 kVP, 10mA, and 0.5mm copper filtration. The dose rate was 320 cGy/min to the midline at a 2-cm depth within the jig at a 35-cm source-to-surface distance. Thermoluminescence dosimetry was used for a liver phantom within the jig as the basis for all corrected dose calculations. Experimental cohorts included untreated controls (no surgery or anesthesia; n = 6), sham-RT (0 Gy, animals received laparotomy with liver exposed to air but no radiation; n = 8), 10 Gy (n = 8) and 50 Gy (n = 8) WLI. Liver and plasma samples were collected at 24 hours, ad-lib, after sham treatment or WLI. For performing 10 Gy WBI, anesthetized C57BL/6 mice (n = 12 per group) were exposed to 0 or 10 Gy of whole-body gamma-radiation using a Shepherd^137^Cs gamma-ray irradiator at a dose rate of 236cGy/min following institutional biosafety guidelines and manufacturer’s instructions.

Two doses of WLI, 10 Gy and 50 Gy were chosen based upon the common fractionation scheme used in hypofractionated stereotactic body radiation therapy (SBRT) of the liver. We have previously demonstrated that 50Gy induces RILD in rodents with significant mortality of the animals [15]. Furthermore, we developed a preparative regimen of hepatocyte transplantation using 50Gy hepatic irradiation (HIR) in rodents [24, 25]. Although a single fraction 50 Gy dose will have higher radiobiological effect than 50Gy in five fractions used in clinical SBRT, higher doses of irradiation is required to cause significant normal tissue toxicity in small irradiated volumes of mouse liver. 10 Gy WLI was used as a low dose of radiation that resulted in changes, which were asymptomatic, yet indicated latent radiation injury. In contrast with 10 Gy WLI dose, which is non-lethal, 10 Gy WBI is a lethal dose and induced acute response.

#### Mass spectrometric data generation and analysis

**Fig 1:**
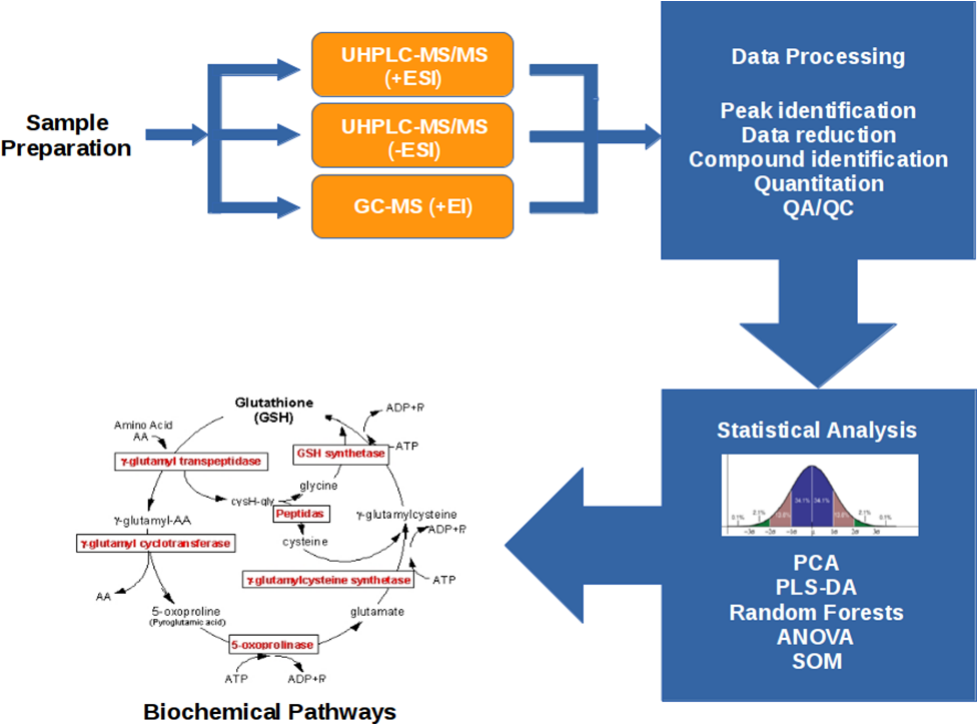
Workflow. This workflow provides an overview of the experimental process including sample preparation, mass spectrometric analysis, peak extraction/identification, compound quantification, statistical analysis for biomarker identification, and mapping of biomarkers to metabolic pathways. Samples were divided into separate groups for gas chromatography/ mass spectrometry (GC/MS) and LC/MS. LC/MS was further divided to examine both positively and negatively charged ions. Following data pre-processing, standard metabolite databases were used to identify metabolites and statistical tests were performed to determine significance of each metabolite in identifying dose-dependent radiation signatures.

Fig 1 contains an overview of the methods used for data generation and analysis, illustrating sample preparation, mass spectrometric analysis, peak extraction/identification and compound quantification, statistical data analysis for biomarker identification and mapping of biomarkers to metabolic pathways. After samples were extracted, they were run under GC/MS and LC/MS conditions [26, 27].

#### Sample Preparation

The sample preparation process was carried out using the automated MicroLab STAR system from the Hamilton Company. Recovery standards were added prior to the first step in the extraction process for QC purposes. After extraction, and protein precipitation, the resulting extract was divided into two equal fractions; for analysis on the GC/MS (Thermo-Finnigan Trace DSQ) and LC/MS (Surveyor HPLC coupled to an LTQ mass spectrometer, Thermo-Finnigan). Samples were placed briefly on a TuboVap (Zymark) to remove the organic solvent. Each sample was then frozen and dried under vacuum. Samples were prepared for the appropriate instrument, either LC/MS or GC/MS.

#### Gas chromatography/Mass Spectrometry (GC/MS)

The samples destined for GC/MS analysis were re-dried under vacuum desiccation for a minimum of 24 hours prior to being derivatized under dried nitrogen using bistrimethyl-silyl-triflouroacetamide (BSTFA). The GC column was 5% phenyl and the temperature ramp was from 40° to 300° C in a 16 minute period. Samples were analyzed on a Thermo-Finnigan Trace DSQ single-quadrupole mass spectrometer using electron impact ionization. The instrument was tuned and calibrated for mass resolution and mass accuracy on a daily basis.

#### Liquid chromatography/Mass Spectrometry for Accurate Mass and MS/MS fragmentation (LC/MS/MS)

The LC/MS portion of the platform was based on a Surveyor HPLC and a Thermo-Finnigan LTQ mass spectrometer, which consisted of an electrospray ionization (ESI) source and linear ion trap (LIT) mass analyzer. Positive and negative ions were monitored within a single analysis by consecutively alternating the ionization polarity of adjacent scans. The vacuum-dried sample was dissolved in 100 μl of an injection solvent that contained five or more injection standards at fixed concentrations. Internal standards were used both to assure injection and chromatographic consistency. The chromatographic system used a binary solvent system delivered as a gradient. Solvent A was water and solvent B was methanol. Both were high purity grade and both contained 0.1% formic acid as a pH stabilizer. The HPLC columns were washed and reconditioned after every injection.

Exact mass determinations were done using a Thermo-Finnigan LTQ-FT mass spectrometer, which had a linear ion-trap (LIT) front end and a Fourier transform ion cyclotron resonance (FT-ICR) mass spectrometer backend. For ions with counts greater than 2 million, accurate mass measurements were performed. Accurate mass measurements were made on the parent ion as well as fragments. The typical mass error was less than 5 ppm. Ions with less than two million counts required a greater amount of effort to characterize. Fragmentation spectra (MS/MS) were typically generated in data dependent manner, but when necessary, targeted MS/MS was employed, such as in the case of lower level signals.

#### Data Analysis, Metabolite Identification and Statistical Calculations

To facilitate visualization of fold changes, all data were normalized to a mean value of 1 for sham-operated (0 Gy-treated) animals. For pair-wise comparisons Welch’s t-tests and/or Wilcoxon’s rank sum tests were used.

#### ANOVA

Statistical analyses were performed on log-transformed data (to account for increases in data variance that occurs as the level of response is increased) using JMP statistical software from SAS Institute, Inc. Two parameters were evaluated when considering statistical significance, namely the p-value and the q-value.

The p-value relates the probability that two comparisons are the same; a low p-value (p<0.05) is generally accepted as a significantly different result. The q-value describes the false discovery rate; a low q-value (q<0.10) is an indication of high confidence in a result. While a higher q-value indicates diminished confidence, it does not necessarily rule out the significance of a result. Compounds with a p-value<0.05 and a q-value<0.10 (false discovery rate) were considered statistically significant. Data were normalized to the relative levels observed in non-irradiated, sham animals.

Spearman’s rank correlation coefficient was used as a non-parametric estimate of the relationship between two metabolite chemical identities, summed over all the experimental groups. For the values of a given metabolite over all experimental groups (sham, 0 Gy, 10 Gy and 50 Gy), $=\;\frac{\sum i\;(xi-\overset{-}{x)}\;(yi-\overset{-}{y)}}{\sqrt{{\sum i\;(xi-\overset{-}{x)}}^{2}{\sum i\;(yi-\overset{-}{y)}}^{2}}}$ where x_i_ is the ranked value (lowest to highest) of a metabolite for the ith sample (total n = 31 for all groups), and y_i_ is the corresponding (dependent) ranked value for another metabolite for that sample in a given group [28, 29].

#### CoolMap heatmap visualization of correlations

CoolMap was used to condense large metabolomic data sets into intuitive heatmap visualizations using pathway ontologies and hierarchical clustering. CoolMap is an interactive visualization software aimed to transform the classic heatmap visualization paradigm. Ontologies were used to reduce original rows and columns of a data table into concepts, in our case metabolic pathways, which made it easier to discover and understand patterns in the data. Spearman’s was used to calculate the correlation between individual metabolites. CoolMap contains aggregator functions (mean, median, average, min, max), which were then used to aggregate the individual existing correlations for metabolites that define (metabolic) pathway ontology. The aggregated values were derived from the corresponding values at the lowest, or the leaf level, for correlated metabolites between pathways.

#### Data Classification

Linear clustering methods such as Principal component analysis (PCA) and orthogonal projections to latent structures discriminant analysis (OPLS-DA) were performed using the SIMCA-13 software package (Umetrics), Random Forest by the method of Breiman [30]. Non-linear clustering method such as The Java Self Organizing map (SOM) Toolbox developed at the Institute of Software Technology and Interactive System at the Vienna University of Technology was used for SOM analysis of the metabolite data [31]. Compound identification was done either by automated comparison to library entries in Metabolon’s internal metabolite library or by spectral matching to the NIST database [27].

## Results

### Data classification and validation framework for determining unique metabolite biomarkers and key pathways affected by liver irradiation

Fig 1 details the workflow used for metabolite extraction, data mining, metabolite identification and validation. For WLI, a total of 595 metabolites were identified between the liver and plasma samples assayed in this study. Full data curation of all mouse liver samples tested in this study yielded 407 chemical entities, 144 were identifiable chemical compounds and 263 represented currently unknown structural identities. By comparison, data curation of all mouse plasma samples tested in this study yielded 347 chemical entities, 129 were identifiable chemical compounds and 218 with currently unknown structural identities. The source(s) of the 595 metabolites are illustrated in Venn diagram format (S1 Fig).

Since the number of animals to power a metabolite study for determinations of radiation damage biomarkers is still yet to be determined for the field, and surveying hundreds of metabolites for a given sample set can be prone to finding spurious correlations, we employed multiple bioinformatic methods to determine the key metabolites that are affected after liver irradiation. Reinforcing identification of key metabolite “hits” was discerned using a decision tree approach for validating and confirming statistically significant metabolite differences between classes, along with Random Forest, PLS-DA, and self organizing maps (SOM). A new unsupervised hierarchical clustering method, CoolMap, was then used for systems integration, relating metabolite “trees” to other individual “trees”, particularly those suggested by the class separation methods, and relate these individual metabolite “trees” to a pathway “forest”, as well as pathways to other pathways, by a correlation defined by the category (ontology) of a group of metabolites, as a biochemical process [32] (see S1 Table).

A decision tree approach was first used to determine consistently altered metabolites for WBI. PCA analysis showed that the sham-treated and 0 Gy-treated groups were separable (S1 Fig), and differences between the 50 Gy and 10 Gy vs. sham treated and 0 Gy groups were used to find consistently altered metabolites between all groups in plasma and liver.

We explored the use of an alternative non-linear unsupervised clustering method, SOM [31], which preserves all metabolite information, in contrast to the limited representation possible with the PCA approach. Using the SOM formalism, one can capture additional non-linear relationships amongst the vector components, and assess their contribution to segmentation apparent on a 2-dimensional map network. The advantage of SOM as a method, is that the component planes of the SOM allows the visual examination of the contribution of each of the metabolites to the cluster separation, emphasizing the utility of the metabolite as a biomarker for radiation injury detection. While ANOVA analysis (S1 and S2 Datasets) indicates significant metabolites, it does not indicate whether the metabolite(s) are important for group separation, although as seen from the subsequent supervised and unsupervised analyses that there is considerable overlap with key metabolites important for group separation. Inspection of a composite of the liver SOMs reveals component planes (S2 Fig) representing metabolites that have a maximal or minimal effect on cluster separation. S3 and S4 Figs illustrate the importance of pentose and purine metabolites to the clustering of specific radiation groups. S5 Fig illustrates the importance of glycolytic metabolites, calcium signaling, and choline metabolism, S6 Fig highlights the importance of bradykinin for plasma class separation, themes reinforced by other classification methods, described below.

**Fig 2:**
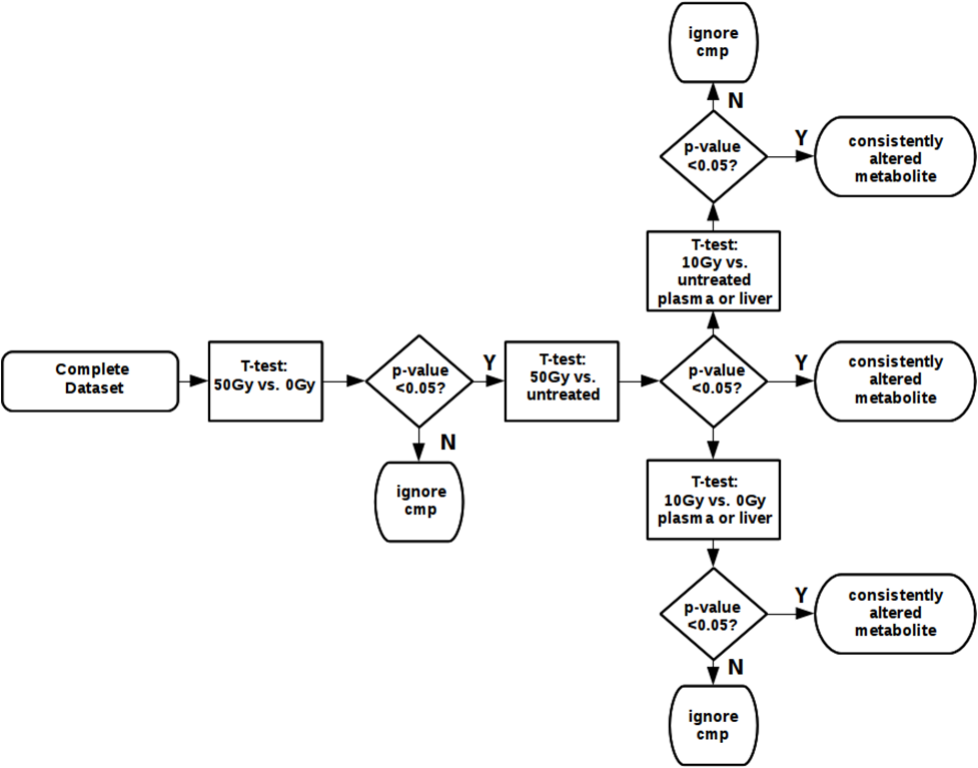
Decision Tree for metabolite validation. The decision tree approach that was used to confirm important metabolite signatures in the WLI experiments is illustrated. Consistently altered metabolites between groups were determined first by comparison of the 50 Gy vs. 0 Gy data sets, then by validation of the metabolite in the 50 Gy vs untreated data sets. If the metabolite was still unconfirmed, it was tested as being significant in the 10Gy vs. 0 Gy plasma or liver data set, or the 10 Gy vs. untreated data sets.

Fig 2 illustrates how the consistently altered metabolites seen in the liver and plasma were determined. The decision tree outlines in Fig 2 was used for comparisons first of the 50 Gy vs. 0 Gy data. Significantly different metabolites, determined by ANOVA (S1 and S2 Datasets), were confirmed by validation of the metabolite as being significant in the 50 Gy vs untreated data sets. If the metabolite was still unconfirmed, it was tested as being significant in the 10 Gy vs. 0 Gy plasma or liver data set, or the 10 Gy vs. untreated data sets. Confirmation of 10 Gy WLI effects with 10 Gy WBI were also determined (S1 Dataset) for known metabolites, and (S2 Dataset) for all metabolites found.

As shown in S2 Dataset, ANOVA analysis on WLI identified 247 significantly changed metabolites in plasma and liver, representing the most significant and consistent metabolomic changes following WLI. For plasma, 74 WLI metabolites were significantly different, 32 with known identities, and 42 “known unknowns”, for which a consistent spectral fingerprint was seen, but could not be related to a metabolite database. At 10 Gy, 38 WLI plasma metabolites were significantly different, 14 of known identity and 24 of unknown identity. Ten of these WLI plasma metabolites were unique for 10 Gy, the rest common to both 50 Gy and 10 Gy.

For liver, 179 significantly changed metabolites were identified, 94 of known identity and 85 “known unknown” metabolites. 53 of the WLI liver metabolites of known identity were seen at 10 Gy and 4 of these were unique, not shared with 50 Gy. 56 of the WLI metabolites of unknown identity were seen at 10 Gy, and 5 of these were unique, not shared with 50 Gy. The rest of the WLI liver metabolites identified were shared between 10 Gy and 50 Gy. Seventeen of the WLI metabolites of known identity were validated as being seen in both WLI liver and plasma, indicating their possible use as biomarkers (S1 Table) Four of the WLI plasma metabolites were found only at one specific Gy level, however, these metabolites were also seen for WBI (pheyllactic acid, DL-indole-3-lactic acid, trans-4-hydroxyproline, and glycerol) (S1 and S2 Datasets). Of the liver metabolites, 3 were found alone at one specific Gy level, however these were seen at WBI (glucose, n-hexadecanoic acid, and 3-hydroxybutanoic acid).

### Correlation and validation of liver and plasma metabolite biomarkers that are altered by WLI and WBI

The radiation dose dependent correlations between plasma and liver metabolites for whole liver radiation are shown in Table 1, and are a composite for all radiation doses 0, 10 Gy and 50 Gy, indicating their possible utility as RILD biomarkers.

**Table 1:** Radiation dose dependent positive correlations between plasma and liver metabolites for whole liver irradiation for the combined radiation levels of 0, 10 or 50 Gy.

Metabolite	Correlation
Aspartate	0.999
Monopalmitin	0.998
Cysteine	0.998
Isobar-21-includes-gamma-aminobutyryl-L-histidine-L-anserine	0.997
3-indoxyl-sulfate	0.996
Niacinamide	0.992
sn-Glycerol-3-phosphate	0.991
1-methylguanidine	0.989
3-methyl-L-histidine	0.986
Inositol	0.984
pantothenic acid	0.984
Isobar-56-includes-DL-pipecolic acid-1-amino-1-cyclopentanecarboxylic acid	0.969
Proline	0.961
Threonine	0.951
Docosahexaenoic-Acid	0.944
palmitoleic acid	0.938
Creatinine	0.937
Sorbitol	0.923
Allantoin	0.894
3-hydroxybutanoic acid	0.880
D-glucose	0.835
o-phosphoethanolamine	0.831
Choline	0.827
2'-deoxyuridine	0.805
glyceric acid	0.795
adenosine-5-monophosphate	0.794
Nonanoate	0.793
Serine	0.792
Cholesterol	0.723
Glucarate	0.715
Alanine	0.708

The Spearman’s correlation coefficient, is defined in Methods.

**Fig 3:**
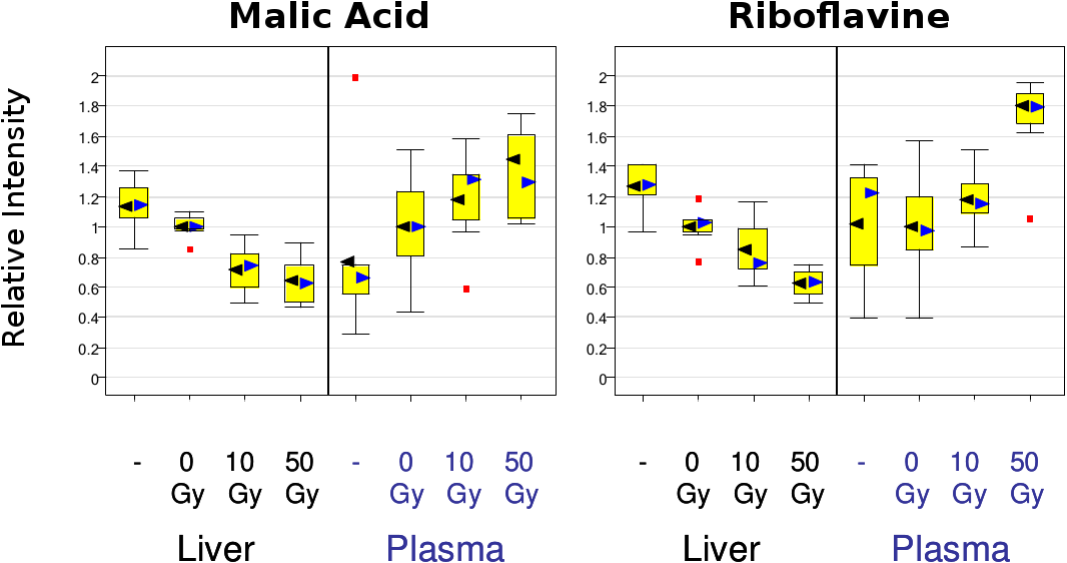
Example of consistent metabolite signatures in liver and plasma. Malic acid and riboflavine are shown as two examples of metabolites, which showed distinctive, consistent signatures in both liver and plasma samples. Supporting information tables show further hits that were consistently altered post-radiation in either liver, or plasma, or both.

Fig 3 shows malic acid and riboflavine as two examples of metabolites, which show distinctive, consistent signatures in both liver and plasma samples, erhaps showing as they became depleted in liver, they were released into plasma. Malic acid and riboflavine were also seen to be important for class separation, as described in following sections. S1 and S2 Datasets further illustrate the metabolite profiles that were consistently altered post-radiation in either liver, or plasma, or both.

For plasma samples, 44 out of 134 known metabolites were significant and for liver samples, 41 out of 134 metabolites were significant for 10 Gy vs non-irradiated mice. There were 30 common metabolites between liver and plasma, supporting their use as radiation injury biomarkers (Table 2). Plasma biomarkers in common between WBI and WLI included 13 out of 134 known metabolites and predominant among these were microbial metabolites such as 3 indoxyl sulfate, indole-3-lactic acid, phenyllactic acid, pipecolic acid, and hippuric acid, and markers of DNA damage such as 2-deoxyuridine (S1 Dataset). Liver biomarkers in common between WBI and WLI included 10 out of 134 known metabolites and predominant among them were amino acids, sugars and TCA metabolites (fumarate), fatty acids (lineolate, n-hexadecanoic acid) and DNA damage markers (uridine) (S1 Dataset).

**Table 2:** Levels of metabolites changing between 0 Gy and 10 Gy in liver and plasma.

COMPOUND	WBI Plasma 10Gy_0Gy ratio	WBI Liver 10Gy_0Gy ratio
Dimethylglycine	0.82	0.79
Pipecolate	0.78	0.83
phenol sulfate	0.56	0.59
Phenylacetylglycine	0.66	0.52
Isovalerylglycine	0.51	0.63
4-Guanidinobutanoic acid	0.49	0.63
3-indoxyl sulfate	0.6	0.5
Citrulline	0.72	0.82
Urea	0.82	0.79
N-acetyl-L-leucine	0.59	0.58
Fructose	0.77	0.75
Heme	0.56	0.42
trigonelline (N'-methylnicotinate)	0.62	0.68
Niacinamide	0.71	0.88
fumaric acid	0.56	0.82
2-hydroxyglutarate	0.6	0.74
myristoleate (14:1n5)	1.71	2.35
palmitoleate (16:1n7)	1.53	1.65
1-palmitoylglycerophosphoinositol	0.61	0.71
palmitoyl sphingomyelin	1.43	1.34
Inositol	0.68	0.79
Allantoin	0.71	0.79
Cinnamoylglycine	0.57	0.56
equol sulfate	0.56	0.65
homostachydrine	0.65	0.56
Stachydrine	0.71	0.57
N-acetylalanine	0.68	1.21
eicosapentaenoate (EPA; 20:5n3)	0.75	1.69
1-linoleoylglycerol (1-monolinolein)	0.66	4.11
Docosahexaenoic Acid	0.79	1.37

Liver and plasma metabolites are positively correlated, except for N-acetylalanine, EPA, 1-monolinolein and Docosahexaenoic Acid which are negatively correlated.

All biomarkers shown in Table 2 were found to be positively correlated between plasma and liver, except for 4, N-acetylalanine, eicosapentaenoate (EPA; 20:5n3), 1-linoleoylglycerol (1-monolinolein) and Docosahexaenoic Acid, which were inversely correlated.

As shown in S1 Dataset with regard to changes seen in metabolites between 0 Gy and 10 Gy, while there were several metabolites that changed in common between WBL and WLI, the majority changed in opposite directions. For plasma, between 0 Gy and 10 Gy pipecolate, 3 indoxyl sulfate, stachydrine, inositol, 2-deoxyuridine, hippuric acid and phenol sulfate increased for WLI and decreased for WBI, in contrast 2'-deoxyuridine showed similar increases for WBI and WLI.

For liver metabolites 10 out of 134 metabobolites were shared as being changed for WBI and WLI. For WBI vs WLI in response to 10 Gy, tryptophan, valine, sorbitol and uridine were lower for WLI in comparison to WBI, fumarate was comparably changed, and inositol and allantoin were higher for WLI than WBI. For WBI vs WLI, comparing the response to 50 Gy for WLI to the response at 10 Gy for WLI, tyrosine, linoleic acid and n-hexadecanoic acid were lower for WLI at 50 Gy than for WBI at 10 Gy

Supervised learning methods such as Random Forest and PLS-DA were used for the WLI plasma and liver samples to determine metabolites that were key to class separation. Table 3 shows the Random Forest approach correctly classified almost 90% of the liver samples, and 76% of the plasma samples, while Table 4 shows that the PLS-DA classifier correctly classifies 100% of the liver samples and 86% of the plasma samples.

**Table 3:** The Random Forest classifier correctly classifies almost 90% of liver and 76% of plasma samples.

**Liver**	**Predicted Class**				
**Actual Class**	Untreated	0 Gy	10 Gy	50 Gy	Error Rate
Untreated	8	0	0	0	0%
0 Gy	1	5	0	0	17%
10 Gy	0	2	5	0	29%
50 Gy	0	0	0	8	0%
**Plasma**	**Predicted Class**				
**Actual Class**	Untreated	0 Gy	10 Gy	50 Gy	Error Rate
Untreated	5	2	1	0	38%
0 Gy	1	3	2	0	50%
10 Gy	1	0	6	0	14%
50 Gy	0	0	0	8	0%

**Table 4:** PLS-DA classifier correctly classifies 100% of liver samples and 86% of plasma samples.

**Liver**	**Predicted Class**				
**Actual Class**	Untreated	0 Gy	10 Gy	50 Gy	Error Rate
Untreated	8	0	0	0	0%
0 Gy	0	6	0	0	0%
10 Gy	0	0	7	0	0%
50 Gy	0	0	0	8	0%
**Plasma**	**Predicted Class**				
**Actual Class**	Untreated	0 Gy	10 Gy	50 Gy	Error Rate
Untreated	7	1	0	0	13%
0 Gy	1	3	2	0	50%
10 Gy	0	0	7	0	0%
50 Gy	0	0	0	8	0%

**Fig 4:**
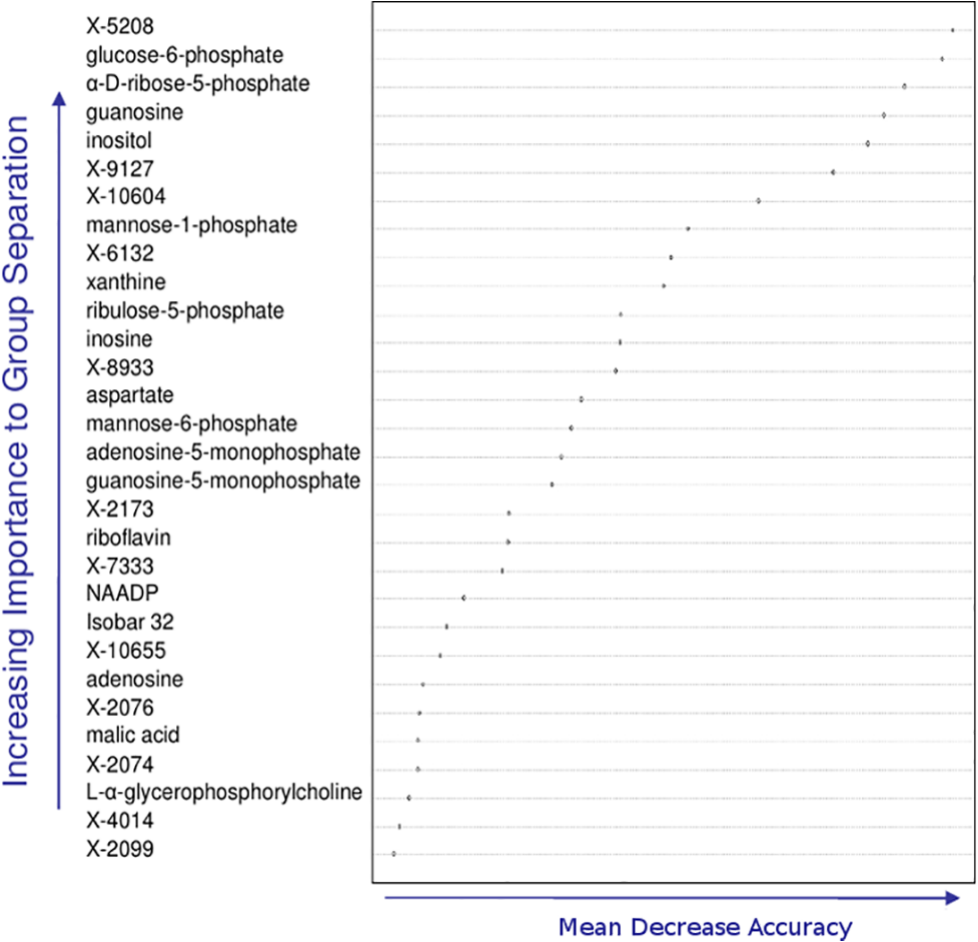
Liver metabolites key to group separation predicted by Random Forest classification. The top 30 liver metabolites important for increasing class separation as determined by the Random Forest approach. Key among these are metabolites representing pentose cycle and related glycolytic metabolites (glucose-6-P, mannose-6P, mannose-1P, ribose-5-P, ribulose-5-P) and purine metabolism (inosine, guanosine, adenosine, xanthine, adenosine-5-P, guanosine-5-P) (see text).

Random Forest analysis on liver samples (Fig 4) showed high importance of pentose cycle and related glycolytic metabolites (glucose-6-P, ribose-5-P, mannose-1-P and mannose-6-P) and purine metabolism (inosine, guanosine, adenosine, xanthine, adenosine-5-P, guanosine-5-P) for class separation, as well as physiological relevance as radiation damage biomarkers (see Discussion). Energy metabolism, calcium signaling and choline metabolism may also have physiological importance as well as being important for class separation, reflected in the identification of riboflavin/malate/aspartate, inositol/NAADP, and glycerophosphorylcholine, respectively (see Discussion).

**Fig 5:**
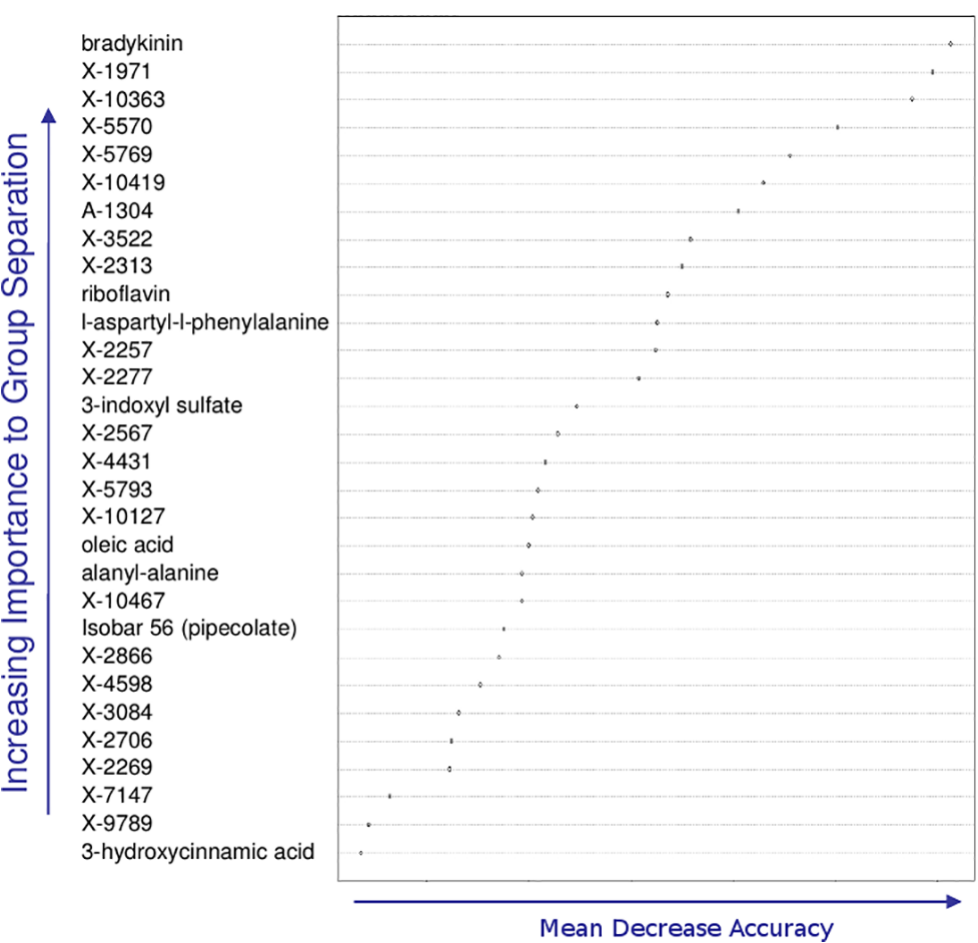
Plasma metabolites key to group separation predicted by Random Forest classification. The top 30 plasma metabolites important for increasing class separation as determined by the Random Forest approach. Bradykinin was seen as most important for class separation, however, smaller peptides also showed class discrimination, such as L-aspartyl-L-phenylalanine, and alanyl-alanine. Energy metabolism, as reflected by the presence of riboflavin as a biomarker, was indicated. Metabolites suggesting multiple organ interactions, i.e. kidney/liver/GI tract/microbiome were indicated (see text).

Random Forest analysis on plasma samples (Fig 5) showed bradykinin as most important for class separation. Smaller peptides also showed class discrimination, such as L-aspartyl-L-phenylalanine, and alanyl-alanine. Energy metabolism, as reflected by the presence of riboflavin, was indicated. Metabolites such as 3-indoxyl sulfate suggested the involvement of the GI tract/microbibiome and by the detection of 3-hydroxycinnamic acid. Oleic acid may signify the affect of radiation to decrease feeding, a biomarker for lipolysis (see Discussion)

**Fig 6:**
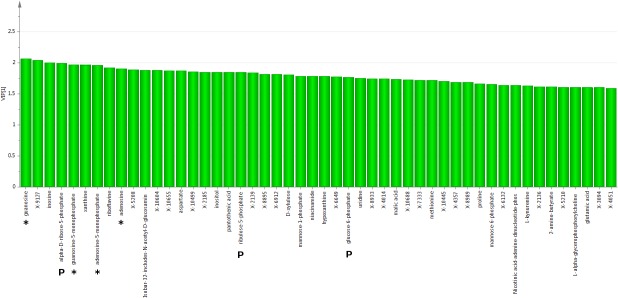
Liver metabolites key to group separation predicted by PLS-DA assessed using variable influence on projections (VIPs). Liver PLS-DA VIPs identified potential biomarkers and exhibited strong overlap with metabolites important for classification using the Random Forest approach. VIP values greater than 1 delineated metabolites most important for cluster classification. An asterisk indicates metabolites in the purine synthesis pathway while a 'P' indicates the pentose phosphate pathway. Both purines and pentose phosphate metabolites were highly important for liver group classification, reinforcing Random Forest findings, and might be determinants of the liver radiation response.

PLS-DA analysis on liver samples (Fig 6) confirmed the importance of purine metabolites, pentose cycle, energy related metabolites (riboflavin/malate/aspartate), calcium signaling (inositol, NAADP), and microbiome related metabolism (2-amino butyrate, kynurenine). Additional biomarkers identified may be supportive of the above metabolic processes, and include pantothenic acid, xylulose, niacnamide, hypoxanthine, methionine, proline and glutamic acid (see Discussion)

**Fig 7:**
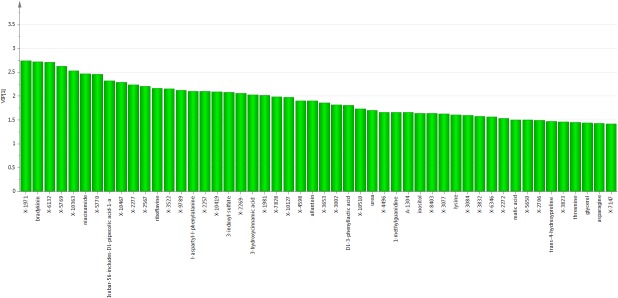
Plasma metabolites key to group separation predicted by PLS-DA assessed using variable influence on projections (VIPs). The most significant known hits for plasma VIPs included: bradykinin, niacinamide, riobflavine, 3-indoxyl-sulfate, and 3-hydroxycinnamic acid. Levels of plasma bradykinin increased more than 25-fold following high dose irradiation. Based on its vasodilator effects, bradykinin may help increase permeability in vasculature damaged by radiation. As in the previous figures, VIP values greater than 1 delineated metabolites most important for cluster classification.

PLS-DA analysis on plasma samples (Fig 7) again showed the importance of bradykinin, and smaller plasma peptides, like L-aspartyl-L-phenyalanine for class separation. Importance of energy metabolism biomarkers for class separation was suggested by detection of niacinamide, riboflavin and malic acid. The identification of inositol in plasma as important for class separation reinforced its potential importance seen in liver metabolic processes (see Discussion). Methylguandine a suspected uremic toxin, and indoxyl sulfate, a kidney/liver/GI tract/microbiome biomarker both were important in class separation. Other microbiome biomarkers such as 3-hydroxycinnamic acid were also important for class separation. Amino acids and their metabolic products were more evident here than in the Random Forest analysis. Lysine, threonine, asparagine, 3-phenyllactic acid and 4-hydroxyproline were identified as important for class separation. 3-phenyllactic acid is a product of phenylalanine catabolism, and 4-hydroxyproline is produced by hydroxylation of the amino acid proline (see Discussion). Glycerol was identified only at 50 Gy, lacking confirmation according to the decision tree approach, but its potential as a lipolysis marker was suggested from another lipolysis marker, oleic acid seen as important in Random Forest plasma analysis. Triglycerides, when broken down in lipolysis, produce glycerol and fatty acids, and oleic acid is a key constituent of triglycerides.

### Identification of correlations within liver and plasma metabolic pathways using CoolMap hierarchical clustering

CoolMap hierarchical clustering analysis condenses large metabolomic data sets into intuitive heatmap visualizations for detection of relationships between metabolites, metabolites and biological processes and between biological processes, out of the entire collection of metabolites in a study (see Methods). The clustering was done on the correlation matrix (Spearman’s) used on the entire collection metabolites, regardless of whatever radiation group they came from, to examine clustering of related groups of metabolites, identifying in some cases previous unknown relationships. This analysis was especially useful for the possible relevance of “known unknown” metabolites, metabolites that consistently appeared across all samples, were identified by a mass/charge (m/z) ratio, a MS/MS fragmentation pattern and a retention time, but did not appear in any known metabolite database (reviewed in Kurland et al [32]). Afterwards, these metabolites could be broken down by classes of radiation exposure to examine their dependence on radiation (see following sections).

**Fig 8:**
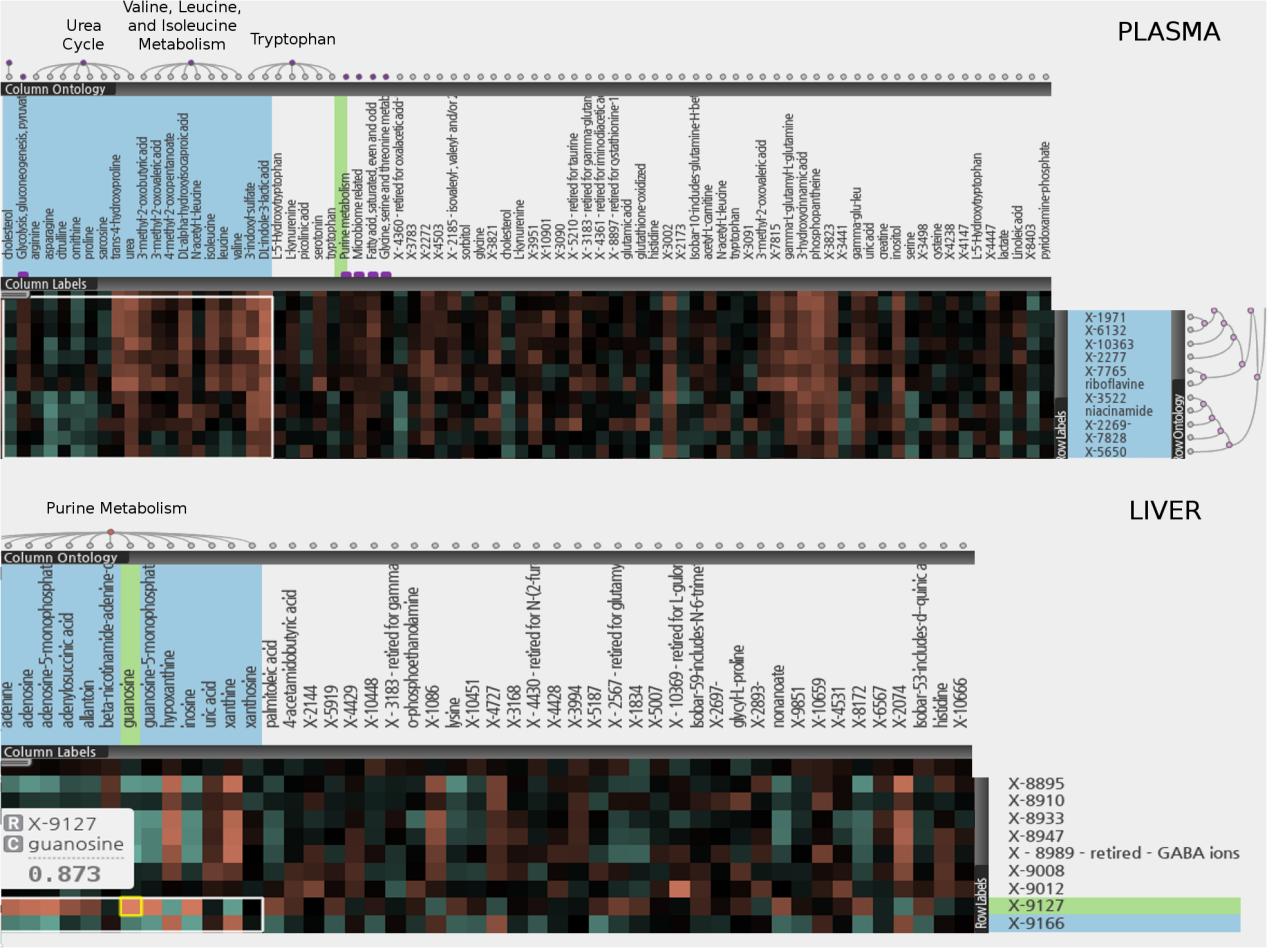
CoolMap and identification of unknown metabolites. CoolMap hierarchical clustering analysis was used for detection of relationships between metabolites, and biological processes in liver or plasma. The top and bottom panels, for plasma and liver, respectively, illustrate on how the “known unknown” metabolites within both plasma and liver samples were correlated with known metabolites that were grouped into ontologies to potentially aid in identification/inference of function. This enabled the relevance of the “known unknowns” seen to be important for class separation by Random Forest and PLS-DA. Top panel: “known unknown” metabolites in plasma are related to urea cycle, branched chain amino acids, tryptophan and microbial metabolites. Bottom panel: “known unknown” metabolites in liver are related to Purine metabolism (see text).

Fig 8 illustrates how the “known unknown” metabolites within both plasma and liver samples can be correlated with known metabolites that were grouped into ontologies to potentially aid in identification/inference of function. This enableed the relevance of the “known unknowns” seen to be important for class separation by Random Forest and PLS-DA. For example, metabolites identified from PLS—DA plasma with significant VIPs, x-1971, x-6132,x-10363, x-2277, x-3522, x-2269, x-7828 and x-5650, shared with Random Forest plasma hits x-1971, x-3522 and x-6132, were part of a block of 9 unknowns which were clustered with riboflavine and niacinamide based on similar profiles. They showed a strong positive correlation with known metabolites in the Valine, Leucine, and Isoleucine Metabolism ontology as well as a general negative correlation with Urea Cycle metabolites, and interestingly, positively with 3 microbial related metabolites, 3-indoxyl sulfate, indole-3-lactic acid and 3-hydroxycinnamic acid. For Liver, PLS-DA VIPs x-9127, x-8895 and x-8933, with x-9127 and x-8933 also identified by Random Forest, there was also a block of 9 unknowns which, (with the exception of X-9127) showed a negative correlation with Purine Metabolism. These plasma and liver CoolMap plot allowed the reinterpretation of plasma and liver PLS-DA and Random Forest plots into showing what biological processes were most significant for class separation, rather than what individual metabolites were important for class separation (see Discussion).

**Fig 9:**
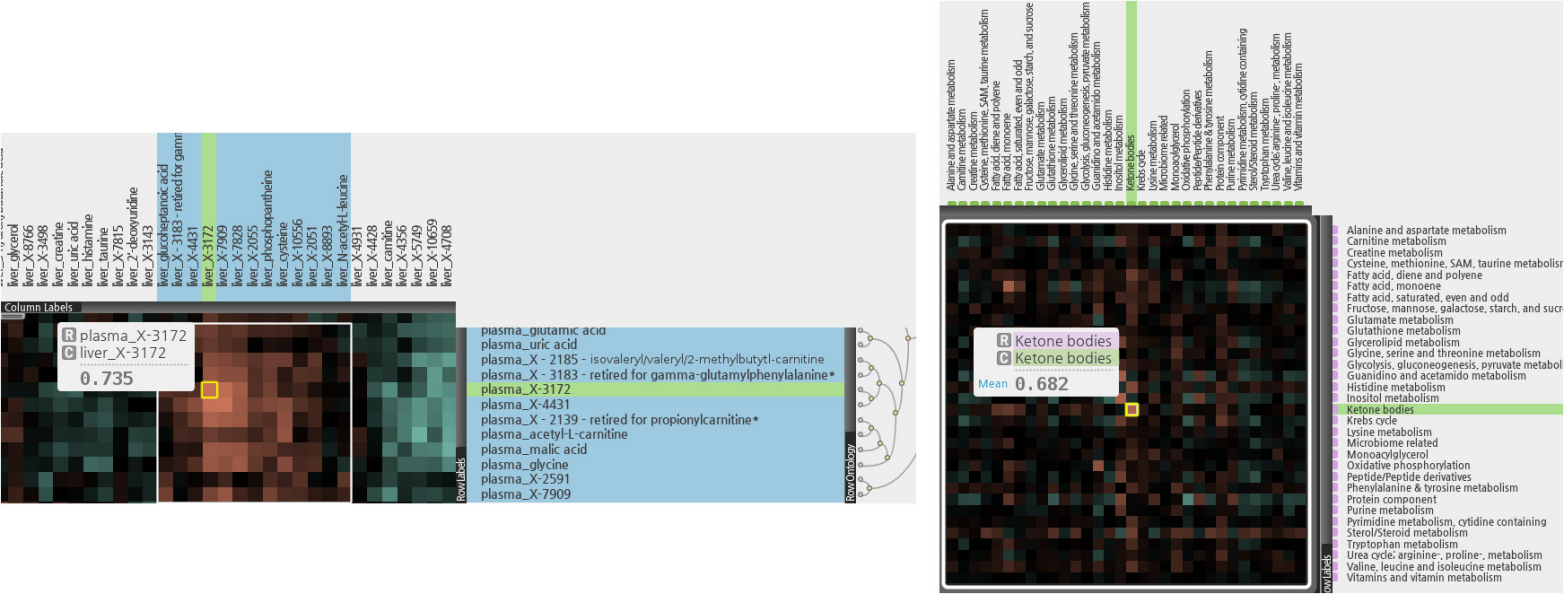
Coolmap and metabolic pathway correlations. Coolmap hierarchical clustering analysis was used for detection of relationships between liver and plasma metabolic processes. Left panel: blocks of highly correlated individual metabolites were identified—this included the same metabolite in liver and plasma (for exampleX-3172). Right panel: Liver and plasma metabolites were grouped into ontologies, and CoolMap was used to examine correlations between different or the same groups of metabolites in liver and plasma. For the same metabolites in liver and plasma there were correlations between liver and plasma ketone bodies, and liver and plasma fatty acid monoenes (see text).

Fig 9 focuses on the liver-plasma correlations. The left panel shows that, blocks of highly correlated individual metabolites were identified—this included the same metabolite in liver and plasma (in this example X-3172). Notable was that with the exception of plasma x-4431, identified as important for class separation for the Random Forest Analysis, and plasma malic acid, identified in PLS-DA, none of the plasma or liver unknown metabolites, despite high correlations, whether positive or negative, were identified in the Random Forest or PLS-DA analyses as important for class separation.

The right panel however shows that when grouped into ontologies, there were some, but not many, substantial correlations between different or the same groups of metabolites in liver and plasma. For the same metabolites in liver and plasma there were correlations between liver and plasma ketone bodies, and liver and plasma fatty acid monoenes, but these are ontological terms which have very few metabolites associated with them. S1 Table shows the members of the ontological terms in both liver and plasma.

### Hepatic pentose cycle metabolites increase in response to liver irradiation

**Fig 10:**
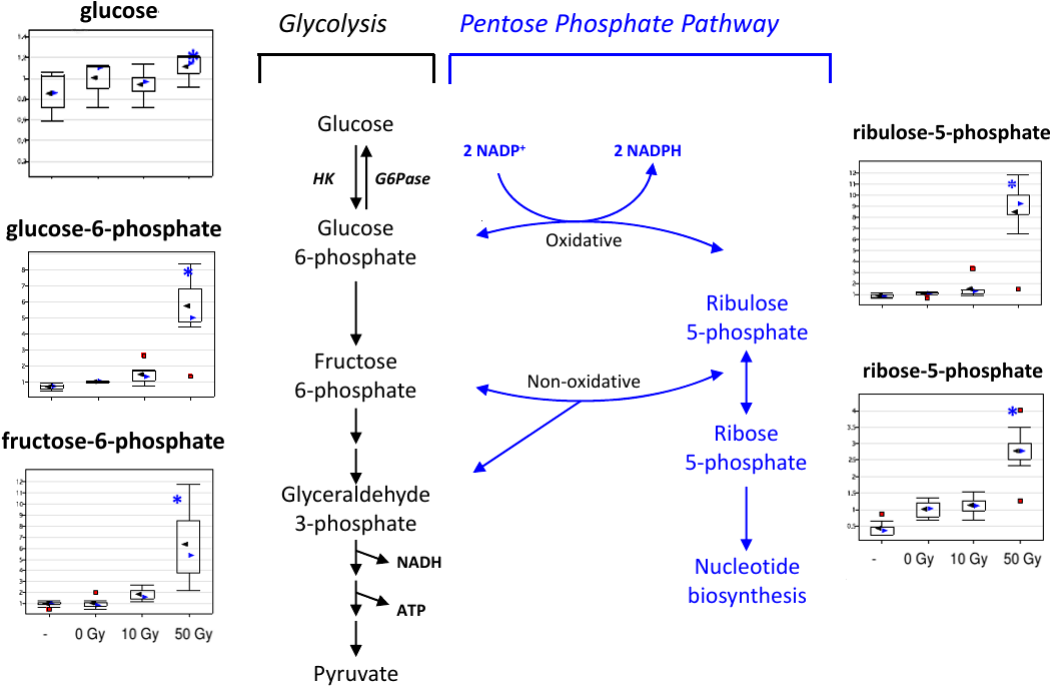
Pentose Phosphate Pathway changes in response to liver irradiation. The pentose phosphate pathway generates NADPH, ribose 5-phosphate, and intermediates of the glycolytic pathway. The NADPH is utilized for reductive pathways, such as fatty acid biosynthesis and the glutathione defense system against injury by reactive oxygen species. Ribose 5-phosphate provides the sugar for nucleotide synthesis. Increased levels of pentose phosphate intermediates may indicate altered glucose and/or nucleotide metabolism.

**Fig 11:**
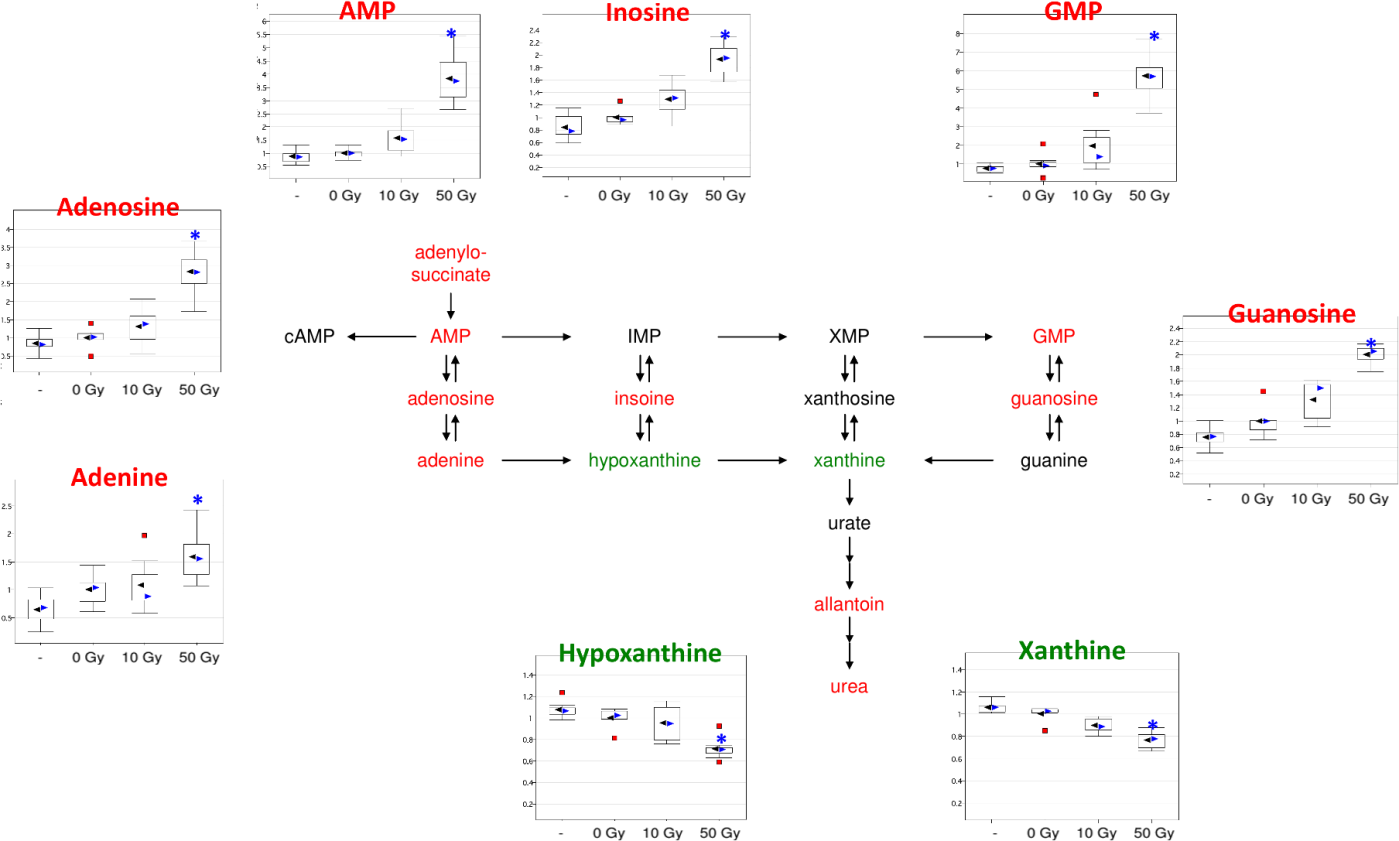
Purine Metabolism changes in response to liver irradiation. A general increase in purine metabolism intermediates may suggest increased breakdown of nucleotides in response to radiation perhaps as a mechanism to support repair after cell injury. Metabolites highlighted in green were decreased after liver irradiation relative to control while metabolites highlighted in red were increased.

The large impact of liver irradiation on hepatic glucose, pentose, TCA, purine, pyrimidine metabolism is illustrated in Figs 10 and 11. Glucose metabolites related to the entry of flux into the oxidative limb of the pentose cycle, glucose-6-phosphate, mannose-6-phosphate, and mannose-1-phosphate, were elevated 3 to 9 fold. It is likely that the changes in pentose phosphate metabolites were due to changes in flux through the oxidative limb, rather than the non-oxidative limb, as ribulose-5-phosphate and ribose-5-phosphate mirrored the changes seen in glucose-6-phosphate. In addition, metabolites downstream of the non-oxidative limb of the pentose cycle were decreased (3-phosphoglycerate, data not shown), supporting that the flux was increased through the oxidative limb of the pentose cycle in response to liver irradiation. The component planes of pentose and purine cycle metabolites showed strong similarities (S3 and S4 Figs), and were important contributors in the SOM segregation of the 50 Gy data from the rest of the data sets.

### Hepatic nucleotide metabolism is affected in response to irradiation

Significant increases (2 to 6 fold) were observed in the AMP and GMP branches of the purine pathway after radiation (Fig 11). By comparison, only two metabolites in the inosine monophosphate (IMP)-inosine-hypoxanthine branch were altered and these changes were not unidirectional (i.e., inosine levels were increased and hypoxanthine and xanthine levels were decreased; Fig 11). The component planes of AMP and GMP branches of the purine metabolic pathway showed strong similarities, and were important contributors in the SOM segregation of the 50 Gy data from the rest of the data sets (S4 Fig).

### Effect of radiation damage on liver and plasma amino acid biomarkers

**Fig 12:**
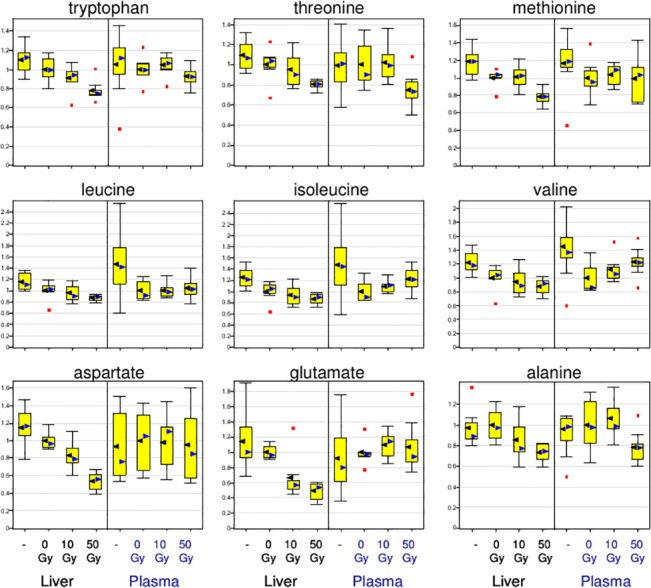
Comparative response of liver and plasma amino acid to irradiation. The relative levels of most amino acids were significantly reduced in mouse liver at 24 h after WLI. Decreased levels of amino acids in the liver after radiation treatment could reflect an increase in protein synthesis or their utilization for energy. Alternatively, decreased levels may result indirectly from an increase in cell volume due to osmotic stress-related changes following irradiation.

As shown in Fig 12, the levels of a number of amino acids were significantly reduced in mouse liver at 24 hours after WLI, including glycine, serine, threonine, alanine, aspartate, glutamate, phenylalanine, tyrosine, tryptophan, isoleucine, leucine, valine, methionine, and proline. Only glutamine and lysine were significantly elevated in mouse liver after treatment with 50 Gy of radiation. Tryptophan, threonine, methonine, leucine, and alanine showed similar changes in plasma and liver.

### Plasma irradiation biomarkers show radiation dose dependence, and a dependence on the microbiome

**Fig 13:**
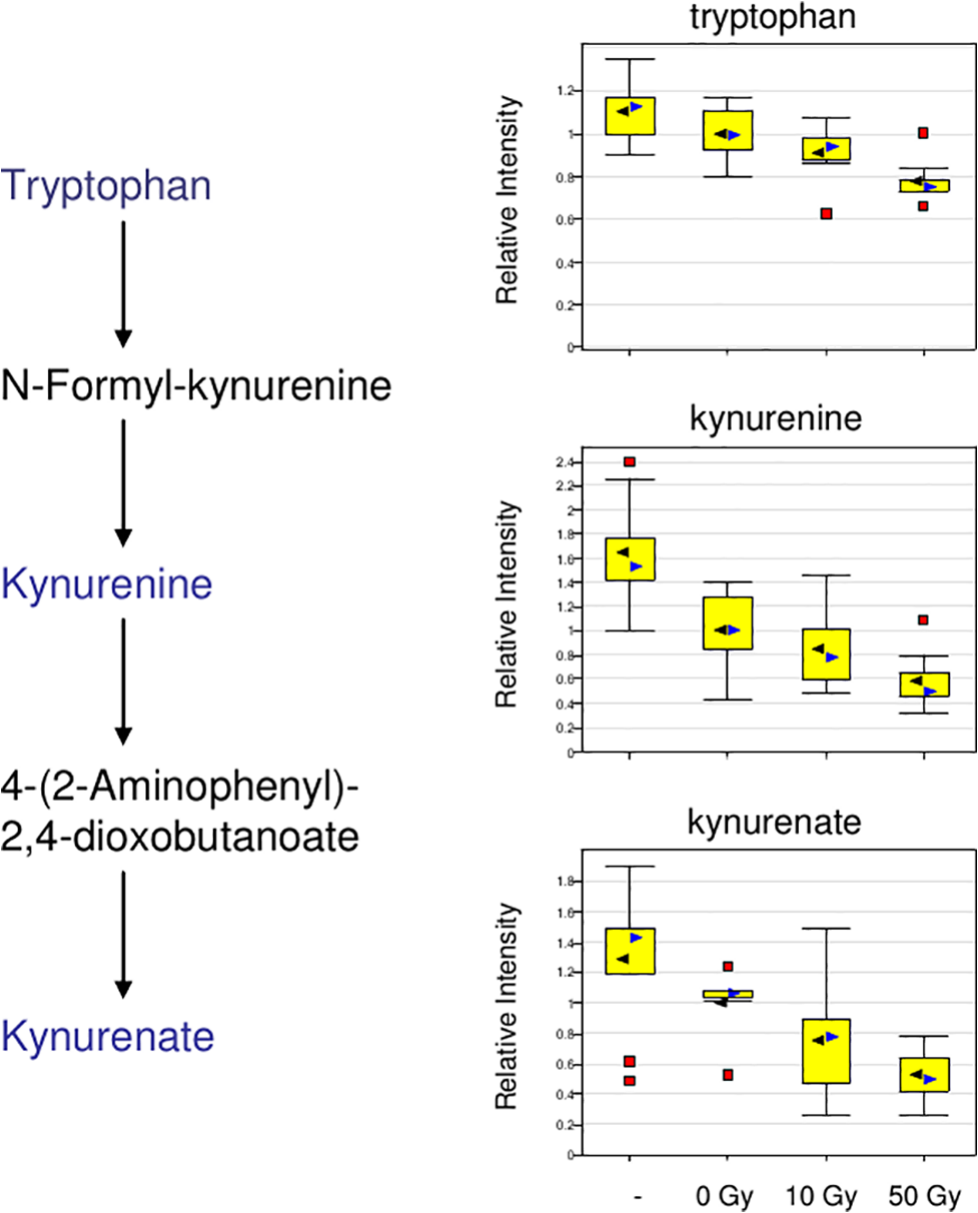
Effects of Liver Irradiation on Tryptophan Metabolism. Normalized levels of tryptophan, kynureinine, kynurenate from liver samples are plotted. Radiation dose-dependent decrease was observed in these metabolites; they were also identified to be important for class separation. Tryptophan and several degradation products—kynurenine and kynurenate—were significantly decreased in liver tissue in response to irradiation.

**Fig 14:**
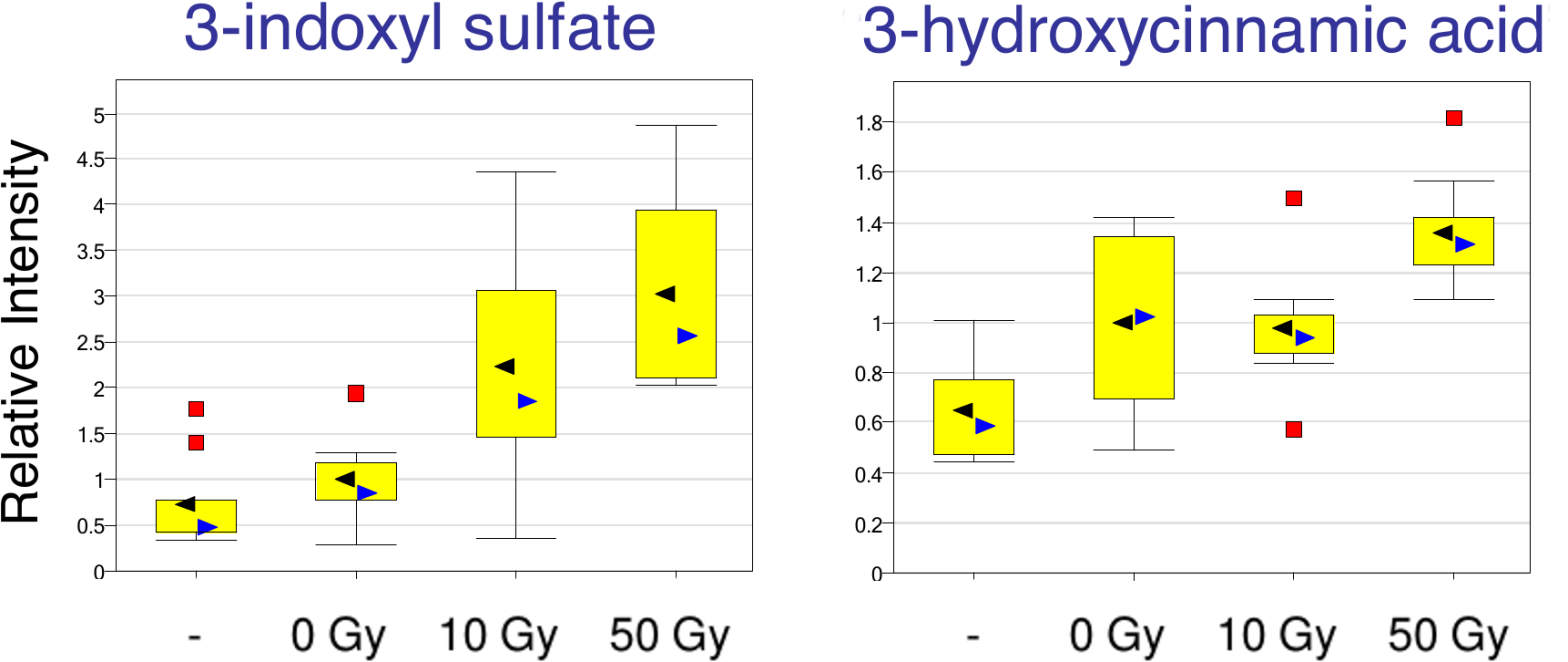
Role of intestinal microbiome in the response to liver Irradiation. Normalized levels of 3-indoxyl-sulfate and 3-hydroxycinnamic acid levels from liver samples are plotted. Radiation dose-dependent increase was observed in these metabolites, indicating possible role of gut microbiome on liver injury. 3-indoxyl-sulfate and 3-hydroxycinnamic acid were both increased in the liver following exposure to radiation. These metabolites are by-products of the intestinal flora, and coupled with those in the Tryptophan pathway point to a possible gut microbiome response to radiation.

Aside from the plasma amino acid biomarkers mentioned, plasma bradykinin showed a ~20-fold increase in response to IR, and plasma indoxyl sulfate, a 3-fold increase in response to HIR at 50 Gy. Both could be important plasma biomarkers of the abdominal irradiation response (see Discussion). Some plasma biomarkers that were correlated with hepatic biomarkers are shown in Fig 9, others in Table 1. Tryptophan and several degradation products—kynurenine and kynurenate—were significantly decreased in liver tissue in response to radiation (Fig 13), while the metabolite indoxyl sulfate was increased (Fig 14). Plasma biomarkers such as indolelactate, hydroxycinnamate and tryptophan metabolites indicated a role for gut microflora in the radiation response, and certainly the importance of indoxyl sulfate as a biomarker was indicated by high correlation between liver and plasma samples at both 10 Gy and 50 Gy (S1 and S2 Datasets). Plasma biomarkers x-1971, x-6132, x-10363, x-2277, x-3522, x-2269, x-7828 and x-5650, important for class separation (see Figs 4–8), were positively correlated with 3 microbial related metabolites, 3-indoxyl sulfate, indole-3-lactic acid and 3-hydroxycinnamic acid (Fig 8) reinforcing the importance of plasma microbial biomarkers for detecting the radiation response. Reinforcing the importance of microbial biomarkers in the radiation injury response were microbial metabolites such as 3 indoxyl sulfate, indole-3-lactic acid, phenyllactic acid, pipecolic acid, and hippuric acid, which were seen in both WBI and WLI plasma.

## Discussion

Metabolomics holds great promise in identifying changes in metabolites that correlate with radiation injury of various organs. Using liver as a model, we demonstrated that while liver contained more identifiable metabolites than plasma in response to liver irradiation, both the liver and plasma showed biomarkers that indicateed tissue-specific causes of radiation injury. By comprehensively analyzing these metabolites and relating them to the biochemical process they are involved in, we demonstrated a change in energy metabolism, calcium signaling, choline metabolism, pentose and purine metabolism and microbiome in response to liver irradiation. This method can be used to provide insight into the overall stress response and the compensatory metabolic pathways involved following radiation injury. Furthermore, the plasma irradiation metabolites showed radiation dose dependence, and a dependence on the microbiome, allowing for the potential use of these metabolites as a biodosimeter and a biomarker for identifying patients at risk of developing RILD.

### Role of Bioinformatics for detecting metabolite markers of radiation damage

We employed multiple bioinformatic methods to validate significant metabolites. Reinforcing decisions regarding metabolite significance were obtained using a decision tree approach for validating and confirming statistically significant metabolite differences found by ANOVA, and then winnowing out the metabolites important for class separation using Random Forest, PLS-DA, and SOM. A new unsupervised hierarchical clustering method, CoolMap, was used to relate unknown metabolites to known metabolites and processes in the first step to determine their possible identities, as well as metabolite processes/pathways affected by radiation to each other. By identifying which unknown metabolites important for class separation were related to known metabolites and biological processes, CoolMap aided the understanding of which biological/metabolic processes were important for class separation, rather than just which metabolites. For example, the bioinformatics approach identified biological processes important for class separation for the effects of liver irradiation that include energy metabolism, calcium signaling, choline metabolism, pentose and purine metabolism and the microbiome.

PLS—DA plasma VIPs x-1971, x-6132,x-10363, x-2277, x-3522, x-2269, x-7828 and x-5650, shared with Random Forest plasma hits x-1971, x-3522 and x-6132, were part of a block of 9 unknowns which were clustered with riboflavine and niacinamide based on similar profiles, and interestingly, positively correlated with 3 microbial related metabolites, 3-indoxyl sulfate, indole-3-lactic acid and 3-hydroxycinnamic acid. *Niacinamide* or vitamin B3 an important compound functioning as a component of the coenzyme NAD, and riboflavin or vitamin B2 as its principal forms in tissues and cells flavin mononucleotide and flavin adenine dinucleotide, both key to energy generation, aiding in the metabolism of fats, carbohydrates, and proteins in the TCA cycle. For Liver, PLS-DA VIPs x-9127, x-8895 and x-8933, with x-9127 and x-8933, also identified by Random Forest, there was also a block of 9 unknowns which, (with the exception of X-9127) showed a negative correlation with Purine Metabolism. Purine metabolism was already indicated as important for class separation by known metabolites identified by Random Forest, PLS-DA and SOM, as was pentose metabolism. Purine and pyrimidine metabolites have been identified as important radiation DNA damage biomarkers in urine [33] [34–36], and future work will relate hepatic damage biomarkers found to urine biomarkers.

Inositol is an isomer of glucose, a cyclic polyalcohol that plays an important role as a second messenger in a cell, as inositol phosphates. In addition, inositol serves as an important component of the structural lipids phosphatidylinositol (PI) and its various phosphates, the phosphatidylinositol phosphate (PIP) lipids. Nicotinic acid adenine dinucleotide phosphate, is a Ca2+-mobilizing second messenger synthesized in response to extracellular stimuli. It is of interest that both NAADP and inositol are important as NAADP, inositol triphosphate and cyclic adenosine diphosphoribose (Cyclic ADP-ribose), binds to and opens Ca^2+^ channels on intracellular organelles, thereby increasing the intracellular Ca^2+^ concentration. Glycerophosphorylcholine (GPCho) and phosphocholine (PCho) are storage forms for choline within the cytosol [37]. The conversion of choline into lipid containing choline storage forms generates a large reserve pool of choline that can be readily accessible at times of high demand,which may explain why rodents and humans do not die from dietary choline deficiency [37].

### Hepatic energy charge is affected after liver irradiation

The decrease in some hepatic amino acids may reflect a decrease in hepatic energy charge. Aspartate and glutamate were dramatically affected in liver and not in plasma (Fig 12). The decrease in liver aspartate and glutamate was apparent even at 10 Gy (Fig 12). These metabolites are related to TCA cycle function, asparate to oxaloacetate via the malate-aspartate shuttle, and glutamate to alpha-ketoglutarate, and their decrease may be a sign of mitochondrial dysfunction/energy depletion. Hepatic malate and fumarate were 30–50% decreased, at 10 Gy and 50 Gy (Fig 12 and S1 Dataset). The hypothesis regarding depletion of hepatic energy charge by upper abdominal irradiation was supported by the most significant of the up-regulated and down-regulated metabolites shown in S1 Dataset. As seen in S1 Dataset, AMP and GMP were greatly up-regulated, AMP 3.8 fold, and GMP 5.7 fold. The decrease in acetyl-L-carnitine may reflect a decrease in acetyl CoA, which, since the liver was sampled in the ad-lib fed state, would also support a decrease in glycolysis.

### Hepatic irradiation biomarkers/metabolites greatly contribute to understanding the overall stress response to liver irradiation

Based on the observed metabolomic changes, several biomarkers have been proposed for the induction of potentially lethal hepatic injury, and interactions of liver metabolism with other organs for the radiation response, most notably the kidney and the GI tract. For liver, several members of the pentose pathway were significantly increased by more than 5-fold including glucose-6-phosphate, mannose-6-phosphate, and mannose-1-phosphate. Glucose-6-phosphate lies on the metabolic crossroads for glycolysis, glycogen metabolism, and the oxidative limb of the pentose phosphate pathway (PPP). The oxidative limb of the PPP generates NADPH for biosynthetic pathways that require large amounts of NADPH, such as fatty acid synthesis, and via reduction of glutathione, suppresses reactive oxygen species (ROS). The oxidative stage of the PPP also generates ribulose-5-phosphate, which was increased 8-fold by WLI. Ribulose-5-phosphate is the precursor for ribose-5- phosphate (which increased nearly 3-fold in liver), the starting point of the non-oxidative stage of the PPP and a precursor for the synthesis of nucleotides. Lastly, the end product of the oxidative stage of the PPP, ribulose-5-phosphate, can be converted to other sugars and sugar phosphates, many of which were elevated in mouse liver following WLI (S1 Dataset). The decrease in acetyl-carnitine and glyceraldehyde-3P, downstream of the pentose cycle, suggested both an increase in carbon flow into the TCA cycle (evidenced by a 5-fold increase in citrate) and increased recirculation through the pentose cycle, involving both oxidative and non-oxidative limbs.

It may be that fatty acid synthesis needs to be increased for irradiation injury repair, and the large upregulation of pentose phosphates may stimulate ChREBP translocation into the nucleus to upregulate glycolysis and fatty acid synthesis. Given the number and magnitude of the changes observed in these carbohydrate metabolism pathways, it is very likely that these metabolites have a large impact on liver function after WLI.

Branches of the purine or pyrimidine metabolism pathways in liver were differentially affected by WLI. Widespread significant changes in purine metabolism pathways were observed in liver samples following WLI, but not in plasma samples. These changes included nucleosides, bases, and other upstream and downstream metabolites. Allantoin, a direct end-product of urate and a proposed oxidative stress marker, was significantly elevated in mouse liver and plasma after WLI. However, it is doubtful that allantoin will represent a useful biomarker for hepatic radiation injury following WLI in humans, as the enzyme (urate oxidase) responsible for conversion of urate to an allantoin precursor is functional in mice but not functional in humans. Overall, it is unclear whether the observed changes in purine metabolism pathways represent mechanisms to support cell growth after radiation injury (i.e. nucleic acid synthesis) or a nucleic acid breakdown response to the radiological insult.

There was a generalized decrease in liver amino acid levels at 24 hours after WLI. Decreases in hepatic amino acid levels can reflect an overall increase in protein synthesis in response to cell damage or other radiation-induced signaling pathways. Alternatively, changes in amino acid levels may indirectly result from an increase in cell volume following irradiation (i.e., an osmotic stress), which would effectively decrease the concentration of amino acids on a per weight basis. An osmotic stress response in response to liver irradiation was supported by the 6-fold increase seen in glycerophosphocholine. Glycerophosphocholine is an abundant renal medullary cytosolic osmolyte that protects renal medullary cells from the high interstitial concentrations of NaCl and urea, normally exposed [38]. In renal medullary cells predominate organic osmolytes are sorbitol, *myo*inositol, glycine betaine and glycerophosphocholine [38]. Sorbitol was downregulated after hepatic irradiation, but insoitol was upregulated, perhaps due to the redirection of glucose metabolism in response to irradiation. Inosine and hypoxanthine are considered stress markers and their differential expression in irradiated liver may suggest very specific functions following radiological insult.

### Plasma Metabolic Biomarkers Diagnostic of Liver Damage and RILD

As summarized in Tables 1 and 2, and S1 Dataset, there is a significant correlation between changes in known liver and plasma metabolites at 0 Gy, 10 Gy and 50 Gy, for WLI, and for the changes between 0 Gy and 10 Gy for WBI. Plasma yielded biomarkers that were radiation dose dependent, within a “window” of time key for radiation exposure diagnosis, suggesting a plasma radiation diagnostic panel may be formulated. S1 and S2 Datasets detail the comparison of associations between plasma and liver metabolites with radiation dose for both WBI and WLI. There were several plasma metabolites that changed in common between WBL and WLI, the majority changed in oppositie directions. For plasma, between 0 Gy and 10 Gy pipecolate, 3 indoxyl sulfate, stachydrine, inositol, 2-deoxyuridine, hippuric acid and phenol sulfate increased for WLI and decreased for WBI, in contrast 2'-deoxyuridine showed similar increases for WBI and WLI.

Bradykinin and 3-indoxyl-sulfate were significantly increased in plasma but not in liver after 50 Gy WLI. Bradykinin is a nine amino acid peptide that functions as a potent vasodilator and a central inflammatory mediator, stimulating an increase in reactive oxygen species generation, like nitric oxide. Our metabolomic data suggests that circulating levels of bradykinin may represent a biomarker for hepatic injury following high-dose radiation treatment, and could result from either increased precursor release (kininogen) from the liver secondary to radiation damage, and/or increased kallikrein activity, fostering bradykinin formation. 3-indoxyl-sulfate is a metabolite formed from the combined interaction of the liver, kidney and GI microbiome. Another indicator of radiation induced kidney damage, perhaps as a result of clearance of the metabolic stress products caused by liver irradiation, is methylguanidine, which accumulates in renal failure [39, 40]. Methylguanidine is synthesized from creatinine concomitant with the synthesis of hydrogen peroxide from endogenous substrates in peroxisomes [41].

Predominant metabolites for plasma biomarkers in common between WBI and WLI were microbial metabolites such as 3 indoxyl sulfate, indole-3-lactic acid, phenyllactic acid, pipecolic acid, and hippuric acid, clearly indicating that tryptophan metabolites were altered by radiation. A number of cell types including macrophages and liver cells have been shown to metabolize tryptophan to kynurenine and kynurenate. It is not clear whether the decrease in hepatic tryptophan metabolites plays a significant biological role in liver cells under the tested conditions. However, a subset of enteric bacteria express tryptophanase, an enzyme that converts tryptophan to indoles. Indoxyl sulfate, which was increased in plasma 3 fold at 50 Gy and 2 fold at 10 Gy, is a known nephrotoxin that accumulates in the blood of patients suffering from chronic kidney failure [42], it arises from hepatic transformation of the bacterial metabolite indole, suggesting that this metabolite could represent an interesting biomarker for hepatic injury. Tryptophanase activity derives from only a subset of enteric bacteria [21]. Non-indole-producing bacteria, such as various *Bifidobacterium* species, have been administered as a test probiotic to the dialysis patients to decrease their plasma levels of indoxyl sulfate [43], and probiotic therapy may be useful as an adjunct for amelioration of the effects of RILD.

Phenolic compounds can be generated by the microbiome, and are carbon ring molecules with an available attachment site for sulfate. These compounds are attractive for the purpose of transporting sulfate through the blood stream. A single phenol can perform this function multiple times, and phenols may be responsible for supplying sulfate for detoxifying xenobiotics and bile acids. Phenol sulfate levels in WLI plasma increased 2 fold in response to either 50 Gy or 10 Gy, suggesting this may be part of the RILD compensatory response.

### Comparison of radiation injury by WLI and WBI using metabolomic profiling

When metabolomic comparison were performed for 0 Gy, 10 Gy WLI, 50 WLI and 10 Gy WBI, some of the metabolites showed reversed trend in WBI versus WLI. For instance, indole-3-lactic acid, 3-phenyllactic acid, urea, riboflavin, niacinamide, and gamma-glu-leu, were increased for WLI and decreased for WBI. The reasons for the overall opposite directions for changes for plasma for WBI vs WLI may be related to the compensatory changes caused by direct liver irradiation vs WBI.

It is clear that both WBI and WLI caused mitochondrial damage as fumarate, a key TCA cycle intermediate, decreased for both. Uridine increases for WBI vs WLI, again suggested compensatory mechanisms that replenished nucleotides for DNA repair. The levels of sugars showed a variable response, with sorbitol decreasing in comparison to 0 Gy for both WBI and WLI, but to a much greater decrease for WLI vs WBI and inositol being low for WBI and higher for WLI.

To our knowledge, this is the first comprehensive analysis of metabolomic biomarkers, as early as 24 hours after whole liver irradiation in mice. We demonstrate a comprehensive metabolomic approach, which helped us identify integrative, systemic, and tissue (liver)-specific metabolic signatures that could be successfully used as surrogate biomarkers for early detection of RILD. In future studies, the significance of plasma metabolomic biomarkers to physiological processes will be determined at different radiation doses and time intervals.

## Supporting Information

S1 Dataset Validation of the ANOVA analysis for WLI metabolites of known identity. Significantly different metabolites, determined by ANOVA were confirmed by validation of the metabolite as being significant in the 50 Gy vs untreated data sets. If the metabolite was still unconfirmed, it was tested as being significant in the 10 Gy vs. 0 Gy plasma or liver data set, or the 10 Gy vs. untreated data sets. Shown are the validations for WLI liver metabolites vs WLI plasma metabolites, indicated by X (see Fig 2). Also indicated are metabolites common to WLI plasma metabolites vs WBI plasma that serve as the cross-validation for detection f, and the metabolites common to WLI liver vs WBI liver. NA-not applicable indicates metabolites that are not significant.
(XLS)

S2 Dataset Summary of entire ANOVA analysis. 247 significantly changed metabolites in plasma and liver for WLI. This is a subset of the metabolites shown in S1 Fig, representing only the most significant and consistent metabolomic changes following WLI, and pathway ontologies. Significantly different metabolites, determined by ANOVA were confirmed by validation of the metabolite as being significant in the 50 Gy vs untreated data sets. If the metabolite was still unconfirmed, it was tested as being significant in the 10 Gy vs. 0 Gy plasma or liver data set, or the 10 Gy vs. untreated data sets. These validations are indicated by X. YY indicates metabolites that were found only at one Gy in either liver or plasma, not confirmed from WLI data sets. However, YY metabolites were confirmed from WBI studies. Also shown are WBI metabolites and the validations indicating WBI plasma metabolite significance, and cross validations between WBI liver and plasma. NA-not applicable indicates metabolites that are not significant.
(XLS)

S1 Fig Distribution of Metabolites Identified in this Study. 595 metabolites were identified in both liver and plasma of irradiated and non-irradiated samples, with 407 metabolites detected in the liver and 347 detected in the plasma. 248 metabolites were unique to the liver, 159 metabolites common to plasma and liver, and 188 metabolites were unique to plasma. PCA analysis showed separation of sham and control treated experimental groups. Note: SOM analysis (following figures) showed separation of 50 Gy treated groups from all others.
(TIF)

S2 Fig SOM Component Planes. While the PCA for the liver samples showed good separation, the plasma samples showed greater variability and groups were not clearly delineated. A non-linear Self-Organizing Map (SOM) approach was applied which showed improved separation in both liver and plasma. The component planes visualization for each of the individual metabolites gave an indication of its contribution to the overall clustering on the final trained SOM map.
(TIF)

S3 Fig Component Planes Pentose Phosphate Pathway. Examining the component planes constrained by cluster ownership yielded associations that reinforced the significance of the liver PLS-DA VIPs (pentose phosphate metabolites marked as P in Fig 5). Untreated samples are shown in white, 0 Gy samples are light grey, 10 Gy samples are dark grey, and 50 Gy samples are shown in black. Color indicates the relative strength of the association between each metabolite and each node in the trained map ranging from 0% (red) to 100% (blue).
(TIF)

S4 Fig Component Planes Purine Metabolism. As before, component planes showed significant agreement with the liver PLS-DA VIPs (Fig 5), Untreated samples are shown in white, 0 Gy samples are light grey, 10 Gy samples are dark grey, and 50 Gy samples are shown in black. Color indicates the relative strength of the association between each metabolite and each node in the trained map ranging from 0% (red) to 100% (blue).
(TIF)

S5 Fig Component Planes Liver. Individual component planes for some of the important radiation dosage biomarkers in liver. Untreated samples are shown in white, 0 Gy samples are light grey, 10 Gy samples are dark grey, and 50 Gy samples are shown in black. Color indicates the relative strength of the association between each metabolite and each node in the trained map ranging from 0% (red) to 100% (blue).
(TIF)

S6 Fig Component Planes Plasma. Individual component planes for bradykinin, the most important plasma biomarker for high dosages of radiation, identified also by PLS-DA (Fig 6). Untreated samples are shown in white, 0 Gy samples are light grey, 10 Gy samples are dark grey, and 50 Gy samples are shown in black. Color indicates the relative strength of the association between each metabolite and each node in the trained map ranging from 0% (red) to 100% (blue).
(TIF)

S1 Table Ontologies used for CoolMAp classification. The relationship between metabolites (left column) and their functional significance/metabolic pathway (right column) are defined.
(DOCX)
