# Supplementary figures and images for: Integrative Metabolic Signatures for Hepatic Radiation Injury
Source: PLoS One. 2015 Jun 5;10(6):e0124795. doi: 10.1371/journal.pone.0124795 (PMC4457483; doi:10.1371/journal.pone.0124795)

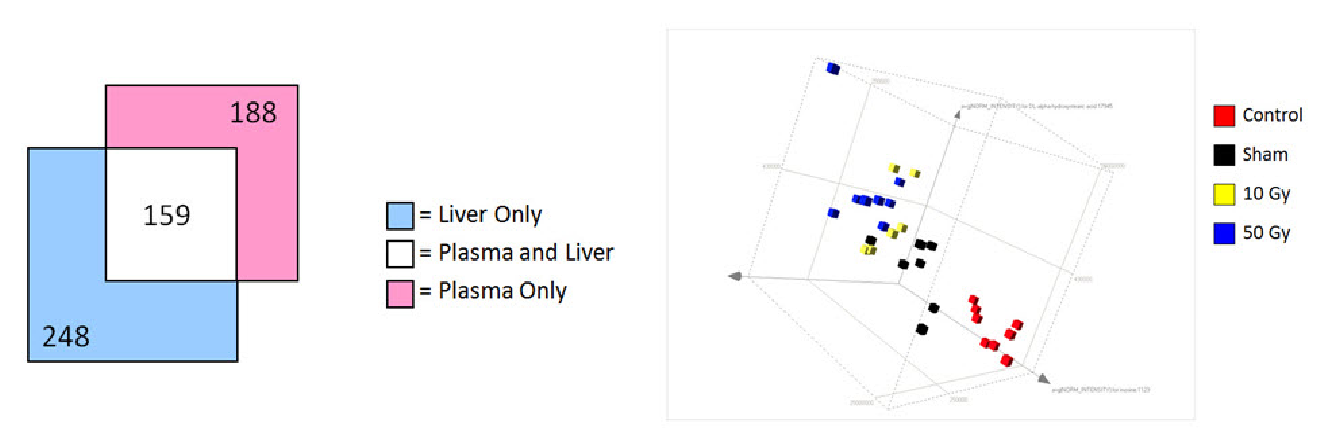

Supplement: S1 Fig — 595 metabolites were identified in both liver and plasma of irradiated and non-irradiated samples, with 407 metabolites detected in the liver and 347 detected in the plasma. 248 metabolites were unique to the liver, 159 metabolites common to plasma and liver, and 188 metabolites were unique to plasma. PCA analysis showed separation of sham and control treated experimental groups. Note: SOM analysis (following figures) showed separation of 50 Gy treated groups from all others. (TIF) [file pone.0124795.s003.tif]

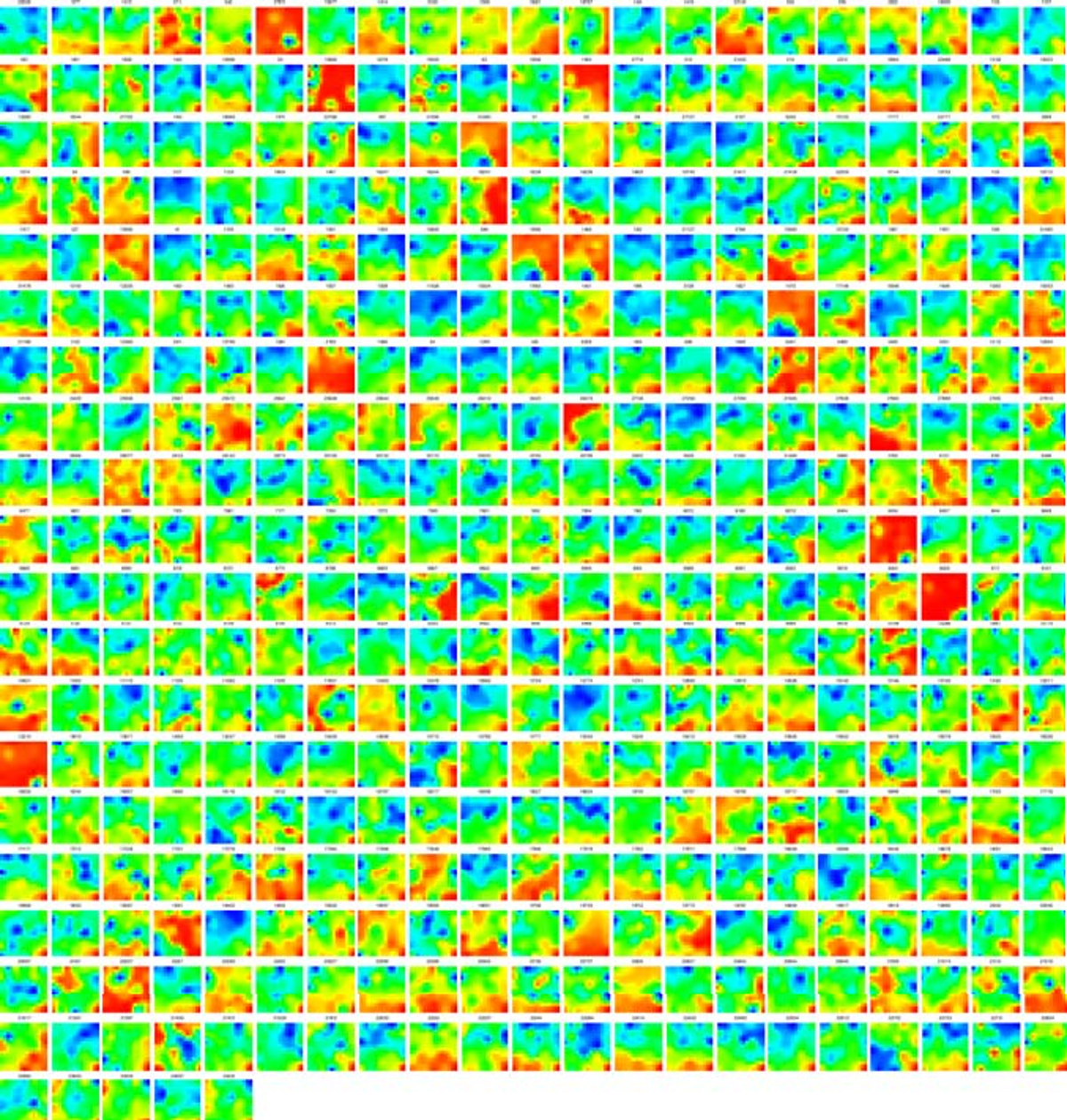

Supplement: S2 Fig — While the PCA for the liver samples showed good separation, the plasma samples showed greater variability and groups were not clearly delineated. A non-linear Self-Organizing Map (SOM) approach was applied which showed improved separation in both liver and plasma. The component planes visualization for each of the individual metabolites gave an indication of its contribution to the overall clustering on the final trained SOM map. (TIF) [file pone.0124795.s004.tif]

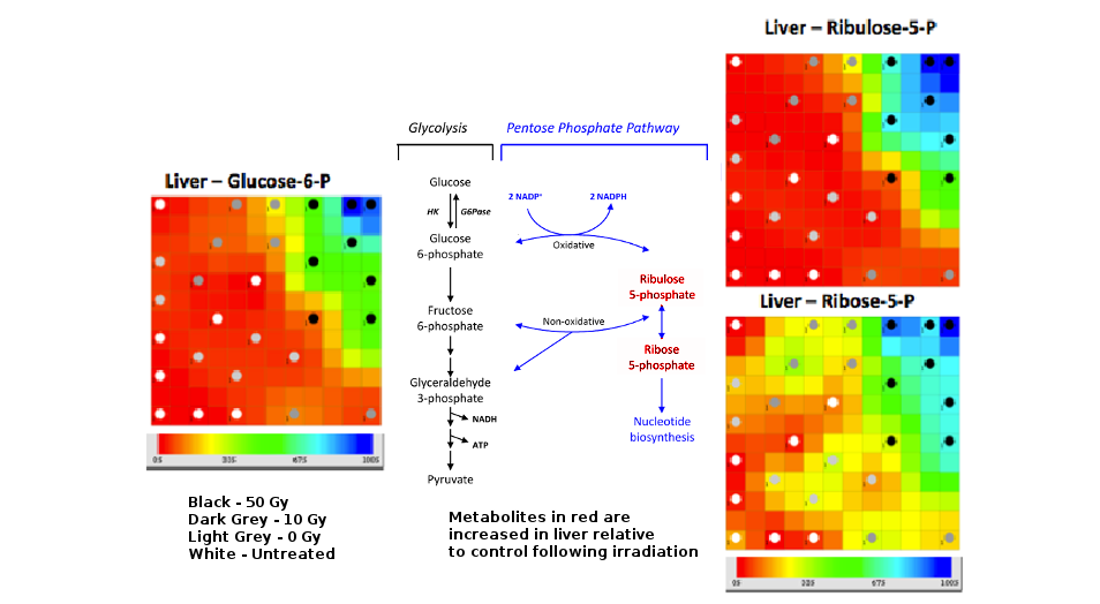

Supplement: S3 Fig — Examining the component planes constrained by cluster ownership yielded associations that reinforced the significance of the liver PLS-DA VIPs (pentose phosphate metabolites marked as P in Fig 5). Untreated samples are shown in white, 0 Gy samples are light grey, 10 Gy samples are dark grey, and 50 Gy samples are shown in black. Color indicates the relative strength of the association between each metabolite and each node in the trained map ranging from 0% (red) to 100% (blue). (TIF) [file pone.0124795.s005.tif]

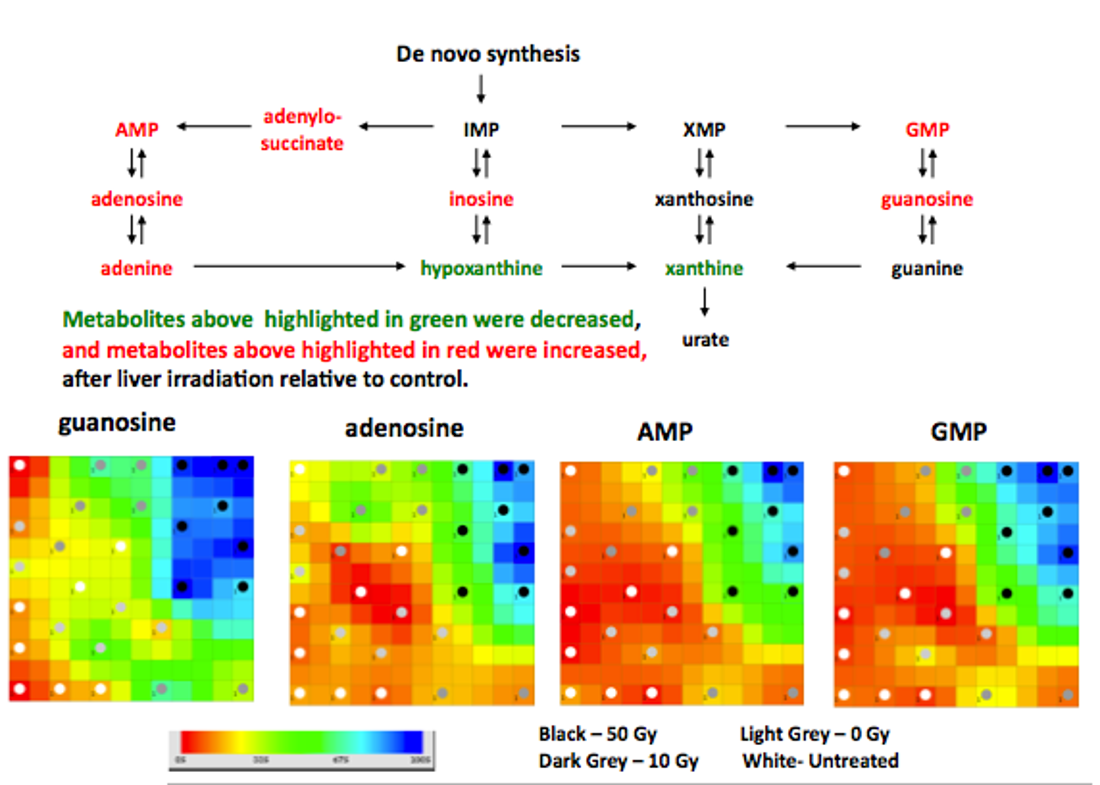

Supplement: S4 Fig — As before, component planes showed significant agreement with the liver PLS-DA VIPs (Fig 5), Untreated samples are shown in white, 0 Gy samples are light grey, 10 Gy samples are dark grey, and 50 Gy samples are shown in black. Color indicates the relative strength of the association between each metabolite and each node in the trained map ranging from 0% (red) to 100% (blue). (TIF) [file pone.0124795.s006.tif]

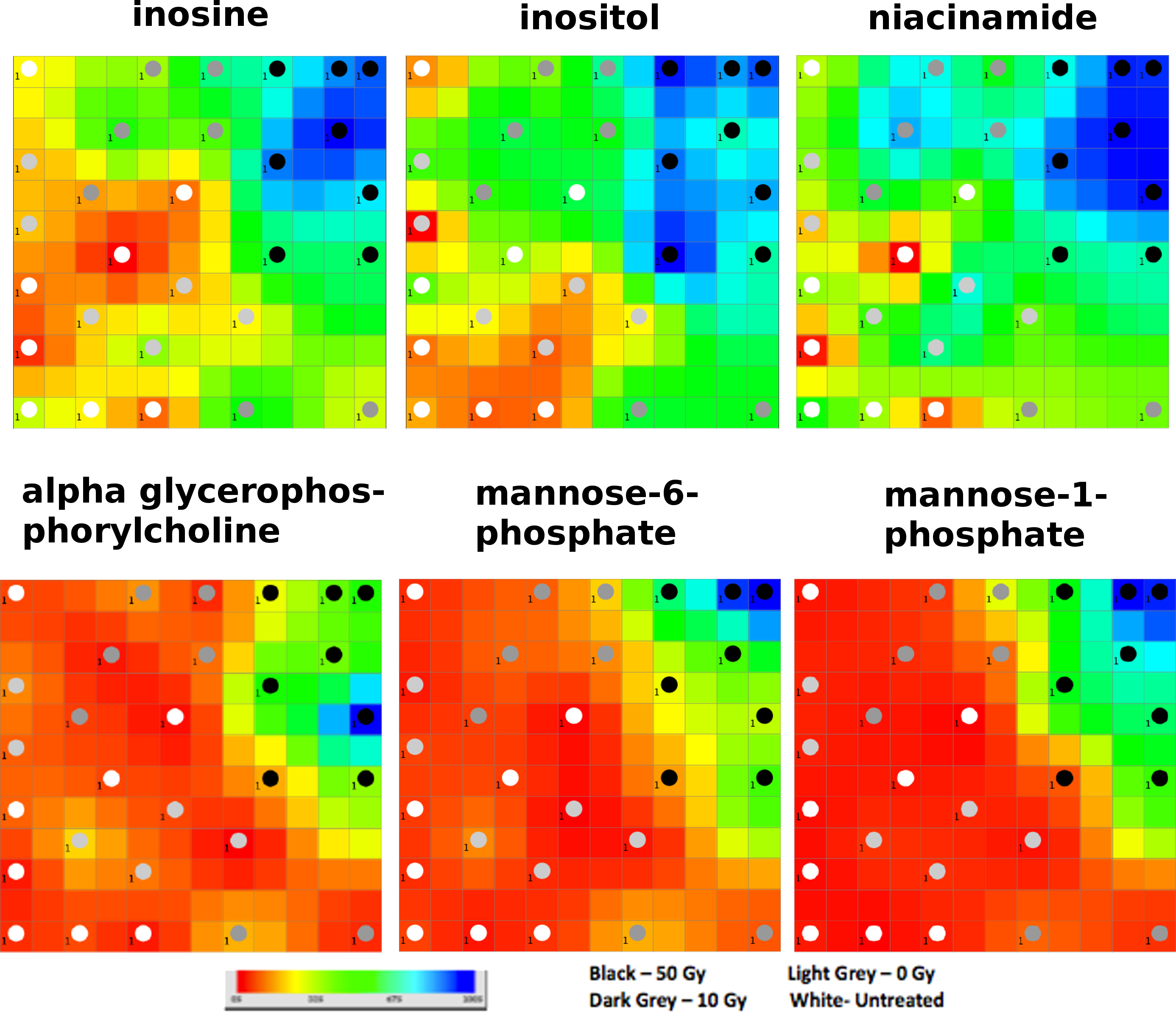

Supplement: S5 Fig — Individual component planes for some of the important radiation dosage biomarkers in liver. Untreated samples are shown in white, 0 Gy samples are light grey, 10 Gy samples are dark grey, and 50 Gy samples are shown in black. Color indicates the relative strength of the association between each metabolite and each node in the trained map ranging from 0% (red) to 100% (blue). (TIF) [file pone.0124795.s007.tif]

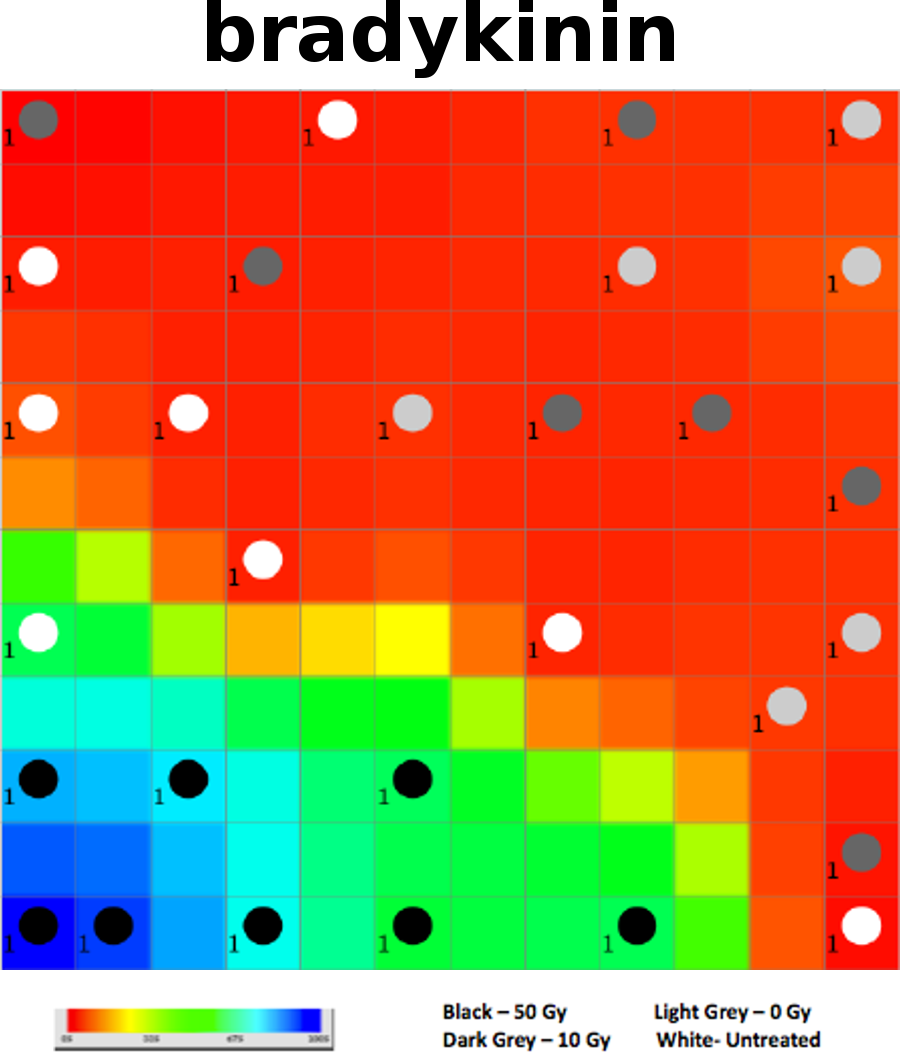

Supplement: S6 Fig — Individual component planes for bradykinin, the most important plasma biomarker for high dosages of radiation, identified also by PLS-DA (Fig 6). Untreated samples are shown in white, 0 Gy samples are light grey, 10 Gy samples are dark grey, and 50 Gy samples are shown in black. Color indicates the relative strength of the association between each metabolite and each node in the trained map ranging from 0% (red) to 100% (blue). (TIF) [file pone.0124795.s008.tif]
