# Supplementary material for: Integrative Metabolic Signatures for Hepatic Radiation Injury
Source: PLoS One. 2015 Jun 5;10(6):e0124795. doi: 10.1371/journal.pone.0124795 (PMC4457483; doi:10.1371/journal.pone.0124795)
Supplement: S1 Table — The relationship between metabolites (left column) and their functional significance/metabolic pathway (right column) are defined. (DOCX) [file pone.0124795.s009.docx]

| **Metabolic Pathways Metabolites** |
| --- |
| \| Alanine and aspartate metabolism \| 3-ureidopropionic acid \|  \|  \| \| --- \| --- \| --- \| --- \| \| Alanine and aspartate metabolism \| alanine \|  \|  \| \| Alanine and aspartate metabolism \| aspartate \|  \|  \| \| Alanine and aspartate metabolism \| beta-alanine \|  \|  \| \| Aminosugars metabolism \| glucosamine-6-sulfate \|  \|  \| \| Aminosugars metabolism \| Isobar-15-includes-alpha-D-glucosamine-1-phosphate-D-glucosamine-6-phosphate \| \| \| \| Aminosugars metabolism \| Isobar-32-includes-N-acetyl-D-glucosamine-N-acetyl-D-mannosamine \| \|  \| \| Aminosugars metabolism \| N-acetyl-D-galactosamine \|  \|  \| \| Aminosugars metabolism \| N-acetyl-D-mannosamine \|  \|  \| \| Beta Amino Acids and Derivatives \| ritalinic acid \|  \|  \| \| Carnitine metabolism \| acetyl-L-carnitine \|  \|  \| \| Carnitine metabolism \| carnitine \|  \|  \| \| Chemical \| EDTA \|  \|  \| \| Chemical \| glycerol-2-phosphate \|  \|  \| \| Chemical \| hydroxyacetic acid \|  \|  \| \| Chemical \| iminodiacetic acid \|  \|  \| \| Chemical \| Isobar-53-includes-d--quinic acid-D-saccharic acid-1-4-lactone-monohydrate \|  \|  \| \| Chemical \| triethyleneglycol \|  \|  \| \| Creatine metabolism \| creatine \|  \|  \| \| Creatine metabolism \| creatinine \|  \|  \| \| Cysteine, methionine, SAM, taurine metabolism \| cysteine \|  \|  \| \| Cysteine, methionine, SAM, taurine metabolism \| hypotaurine \|  \|  \| \| Cysteine, methionine, SAM, taurine metabolism \| methionine \|  \|  \| \| Cysteine, methionine, SAM, taurine metabolism \| taurine \|  \|  \| \| Fatty acid, diene and polyene \| Docosahexaenoic-Acid \|  \|  \| \| Fatty acid, diene and polyene \| Linoleic acid \|  \|  \| \| Fatty acid, monoene \| oleic acid \|  \|  \| \| Fatty acid, monoene \| palmitoleic acid \|  \|  \| \| Fatty acid, saturated, even and odd \| n-hexadecanoic acid \|  \|  \| \| Fatty acid, saturated, even and odd \| nonanoate \|  \|  \| \| Fatty acid, saturated, even and odd \| octadecanoic acid \|  \|  \| \| Fatty acid, saturated, even and odd \| tetradecanoic acid \|  \|  \| \| Food component/Plant \| tartaric acid \|  \|  \| \| Fructose, mannose, galactose, starch, and sucrose metabolism \| 4-O-beta-galactopyranosyl-D-mannopyranose \|  \|  \| \| Fructose, mannose, galactose, starch, and sucrose metabolism \| erythrose \|  \|  \| \| Fructose, mannose, galactose, starch, and sucrose metabolism \| galactonic acid \|  \|  \| \| Fructose, mannose, galactose, starch, and sucrose metabolism \| Isobar-9-includes-sucrose-beta-D-lactose-D-Maltose-and-others \|  \|  \| \| Fructose, mannose, galactose, starch, and sucrose metabolism \| Isobar-30-includes-maltotetraose-stachyose \|  \|  \| \| Fructose, mannose, galactose, starch, and sucrose metabolism \| maltose \|  \|  \| \| Fructose, mannose, galactose, starch, and sucrose metabolism \| mannose \|  \|  \| \| Fructose, mannose, galactose, starch, and sucrose metabolism \| mannose-1-phosphate \|  \|  \| \| Fructose, mannose, galactose, starch, and sucrose metabolism \| mannose-6-phosphate \|  \|  \| \| Fructose, mannose, galactose, starch, and sucrose metabolism \| sorbitol \|  \|  \| \| Glutamate metabolism \| glutamic acid \|  \|  \| \| Glutamate metabolism \| glutamine \|  \|  \| \| Glutamate metabolism \| Isobar-10-includes-glutamine-H-beta-ala-gly-OH-1-methylguanine-H-Gly-Sar-OH-lysine \| \| \| \| Glutathione metabolism \| 5-oxoproline \|  \|  \| \| Glutathione metabolism \| glutathione-oxidized \|  \|  \| \| Glutathione metabolism \| glutathione-reduced \|  \|  \| \| Glutathione metabolism \| X-1595-possible-glutathione-metabolite \|  \|  \| \| Glycerolipid metabolism \| choline \|  \|  \| \| Glycerolipid metabolism \| glycerol \|  \|  \| \| Glycerolipid metabolism \| L-alpha-glycerophosphorylcholine \|  \|  \| \| Glycerolipid metabolism \| o-phosphoethanolamine \|  \|  \| \| Glycerolipid metabolism \| sn-Glycerol-3-phosphate \|  \|  \| \| Glycine, serine and threonine metabolism \| beta-hydroxypyruvic acid \|  \|  \| \| Glycine, serine and threonine metabolism \| glycine \|  \|  \| \| Glycine, serine and threonine metabolism \| serine \|  \|  \| \| Glycine, serine and threonine metabolism \| threonine \|  \|  \| \| Glycolysis, gluconeogenesis, pyruvate metabolism \| 1,5-anhydro-D-glucitol \|  \|  \| \| Glycolysis, gluconeogenesis, pyruvate metabolism \| 3-phospho-d-glycerate \|  \|  \| \| Glycolysis, gluconeogenesis, pyruvate metabolism \| D-glucose \|  \|  \| \| Glycolysis, gluconeogenesis, pyruvate metabolism \| glucose-6-phosphate \|  \|  \| \| Glycolysis, gluconeogenesis, pyruvate metabolism \| glyceric acid \|  \|  \| \| Glycolysis, gluconeogenesis, pyruvate metabolism \| glycerol \|  \|  \| \| Glycolysis, gluconeogenesis, pyruvate metabolism \| lactate \|  \|  \| \| Glycolysis, gluconeogenesis, pyruvate metabolism \| pyruvate \|  \|  \| \| Glycolysis, gluconeogenesis, pyruvate metabolism \| sn-Glycerol-3-phosphate \|  \|  \| \| Guanidino and acetamido metabolism \| 1-methylguanidine \|  \|  \| \| Guanidino and acetamido metabolism \| 4-acetamidobutyric acid \|  \|  \| \| Guanidino and acetamido metabolism \| 4-Guanidinobutanoic acid \|  \|  \| \| Hemoglobin and porphyrin metabolism \| biliverdin \|  \|  \| \| Histidine metabolism \| 1-methyl-L-histidine \|  \|  \| \| Histidine metabolism \| 3-methyl-L-histidine \|  \|  \| \| Histidine metabolism \| histamine \|  \|  \| \| Histidine metabolism \| histidine \|  \|  \| \| Inositol metabolism \| inositol \|  \|  \| \| Inositol metabolism \| inositol-1-phosphate \|  \|  \| \| Intracellular calcium signaling \| Nicotinic acid-adenine-dinucleotide-phosphate-sodium-salt \|  \|  \| \| Ketone bodies \| 3-hydroxybutanoic acid \|  \|  \| \| Krebs cycle \| aconitate \|  \|  \| \| Krebs cycle \| alpha-keto-glutarate \|  \|  \| \| Krebs cycle \| citrate \|  \|  \| \| Krebs cycle \| fumaric acid \|  \|  \| \| Krebs cycle \| isocitrate \|  \|  \| \| Krebs cycle \| malic acid \|  \|  \| \| Lysine metabolism \| alpha-amino-adipate \|  \|  \| \| Lysine metabolism \| glutarate \|  \|  \| \| Lysine metabolism \| Isobar-56-includes-DL-pipecolic acid-1-amino-1-cyclopentanecarboxylic acid \|  \|  \| \| Lysine metabolism \| lysine \|  \|  \| \| Microbiome related \| 3-hydroxycinnamic acid \|  \|  \| \| Microbiome related \| allantoin \|  \|  \| \| Microbiome related \| glucoheptanoic acid \|  \|  \| \| Microbiome related \| hippuric acid \|  \|  \| \| Microbiome related \| p-hydroxybenzaldehyde \|  \|  \| \| Microbiome related \| Isobar-59-includes-N-6-trimethyl-L-lysine-H-homoarg-OH \|  \|  \| \| Monoacylglycerol \| monopalmitin \|  \|  \| \| Monoacylglycerol \| Stearoylglycerol-monostearin- \|  \|  \| \| Nucleotide sugars, pentose metabolism \| gluconic acid \|  \|  \| \| Nucleotide sugars, pentose metabolism \| Isobar-4-includes-Gluconic acid-DL-arabinose-D-ribose-L-xylose-DL-lyxose-D-xylulose-galactonic acid \| \| \| \| Nucleotide sugars, pentose metabolism \| Isobar-24-includes-L-arabitol-adonitol-xylitol \|  \|  \| \| Nucleotide sugars, pentose metabolism \| D-ribose \|  \|  \| \| Nucleotide sugars, pentose metabolism \| alpha-D-ribose-5-phosphate \|  \|  \| \| Nucleotide sugars, pentose metabolism \| ribulose-5-phosphate \|  \|  \| \| Nucleotide sugars, pentose metabolism \| xylitol \|  \|  \| \| Nucleotide sugars, pentose metabolism \| D-xylulose \|  \|  \| \| Oxidative phosphorylation \| phosphate \|  \|  \| \| Peptide/Peptide derivatives \| carnosine \|  \|  \| \| Peptide/Peptide derivatives \| D-alanyl-D-alanine \|  \|  \| \| Peptide/Peptide derivatives \| gamma-glu-leu \|  \|  \| \| Peptide/Peptide derivatives \| gamma-L-glutamyl-L-glutamine \|  \|  \| \| Peptide/Peptide derivatives \| gamma-L-glutamyl-L-tyrosine \|  \|  \| \| Peptide/Peptide derivatives \| glycyl-L-proline \|  \|  \| \| Peptide/Peptide derivatives \| Isobar-10-includes-glutamine-H-beta-ala-gly-OH-1-methylguanine-H-Gly-Sar-OH-lysine \| \| \| \| Peptide/Peptide derivatives \| Isobar-21-includes-gamma-aminobutyryl-L-histidine-L-anserine \|  \|  \| \| Peptide/Peptide derivatives \| l-aspartyl-l-phenylalanine \|  \|  \| \| Peptide/Peptide derivatives \| X-3430-possible-gly-leu-acetyl-lys-ala-val \|  \|  \| \| Phenylalanine & tyrosine metabolism \| DL-3-phenyllactic acid \|  \|  \| \| Phenylalanine & tyrosine metabolism \| Isobar-45-includes-phenylalanine-and-4-2-Dimethylamino-ethyl-phenol \| \|  \| \| Phenylalanine & tyrosine metabolism \| p-hydroxyphenyllactic acid \|  \|  \| \| Phenylalanine & tyrosine metabolism \| thyroxine \|  \|  \| \| Phenylalanine & tyrosine metabolism \| tyrosine \|  \|  \| \| Polypeptide \| bradykinin \|  \|  \| \| Protein component \| phosphopantheine \|  \|  \| \| Purine metabolism \| 1-methyladenine \|  \|  \| \| Purine metabolism \| adenine \|  \|  \| \| Purine metabolism \| adenosine \|  \|  \| \| Purine metabolism \| adenosine-5-monophosphate \|  \|  \| \| Purine metabolism \| adenylosuccinic acid \|  \|  \| \| Purine metabolism \| allantoin \|  \|  \| \| Purine metabolism \| beta-nicotinamide-adenine-dinucleotide \|  \|  \| \| Purine metabolism \| guanosine \|  \|  \| \| Purine metabolism \| guanosine-5-monophosphate \|  \|  \| \| Purine metabolism \| hypoxanthine \|  \|  \| \| Purine metabolism \| inosine \|  \|  \| \| Purine metabolism \| Isobar-10-includes-glutamine-H-beta-ala-gly-OH-1-methylguanine-H-Gly-Sar-OH-lysine \| \| \| \| Purine metabolism \| uric acid \|  \|  \| \| Purine metabolism \| xanthine \|  \|  \| \| Purine metabolism \| xanthosine \|  \|  \| \| Pyrimidine metabolism, cytidine containing \| 2'-deoxyuridine \|  \|  \| \| Pyrimidine metabolism, cytidine containing \| cytidine \|  \|  \| \| Pyrimidine metabolism, cytidine containing \| cytidine-5-monophosphate \|  \|  \| \| Pyrimidine metabolism, cytidine containing \| orotic acid \|  \|  \| \| Pyrimidine metabolism, cytidine containing \| orotidine-5-phosphate \|  \|  \| \| Pyrimidine metabolism, cytidine containing \| thymidine \|  \|  \| \| Pyrimidine metabolism, cytidine containing \| uracil \|  \|  \| \| Pyrimidine metabolism, cytidine containing \| uridine \|  \|  \| \| Pyrimidine metabolism, cytidine containing \| X-2035-Possible-5-methyl-deoxycytidine-monophosphate \|  \|  \| \| Sterol/Steroid metabolism \| cholesterol \|  \|  \| \| Sterol/Steroid metabolism \| isopentenyl-diphosphate \|  \|  \| \| Trisaccharide \| Isobar-31-includes-1-kestose-maltotriose-melezitose \|  \|  \| \| Tryptophan metabolism \| 3-indoxyl-sulfate \|  \|  \| \| Tryptophan metabolism \| DL-indole-3-lactic acid \|  \|  \| \| Tryptophan metabolism \| L-5-Hydroxytryptophan \|  \|  \| \| Tryptophan metabolism \| L-kynurenine \|  \|  \| \| Tryptophan metabolism \| picolinic acid \|  \|  \| \| Tryptophan metabolism \| serotonin \|  \|  \| \| Tryptophan metabolism \| tryptophan \|  \|  \| \| Urea cycle; arginine-, proline-, metabolism \| arginine \|  \|  \| \| Urea cycle; arginine-, proline-, metabolism \| asparagine \|  \|  \| \| Urea cycle; arginine-, proline-, metabolism \| citrulline \|  \|  \| \| Urea cycle; arginine-, proline-, metabolism \| Isobar-5-includes-asparagine-ornithine-gly-gly \|  \|  \| \| Urea cycle; arginine-, proline-, metabolism \| N-acetyl-L-glutamate \|  \|  \| \| Urea cycle; arginine-, proline-, metabolism \| ornithine \|  \|  \| \| Urea cycle; arginine-, proline-, metabolism \| proline \|  \|  \| \| Urea cycle; arginine-, proline-, metabolism \| sarcosine \|  \|  \| \| Urea cycle; arginine-, proline-, metabolism \| trans-4-hydroxyproline \|  \|  \| \| Urea cycle; arginine-, proline-, metabolism \| urea \|  \|  \| \| Valine, leucine and isoleucine metabolism \| 3-methyl-2-oxobutyric acid \|  \|  \| \| Valine, leucine and isoleucine metabolism \| 3-methyl-2-oxovaleric acid \|  \|  \| \| Valine, leucine and isoleucine metabolism \| 4-methyl-2-oxopentanoate \|  \|  \| \| Valine, leucine and isoleucine metabolism \| DL-alpha-hydroxyisocaproic acid \|  \|  \| \| Valine, leucine and isoleucine metabolism \| isoleucine \|  \|  \| \| Valine, leucine and isoleucine metabolism \| leucine \|  \|  \| \| Valine, leucine and isoleucine metabolism \| N-acetyl-L-leucine \|  \|  \| \| Valine, leucine and isoleucine metabolism \| valine \|  \|  \| \| Vitamins and vitamin metabolism \| alpha-tocopherol \|  \|  \| \| Vitamins and vitamin metabolism \| glucarate \|  \|  \| \| Vitamins and vitamin metabolism \| methylcobalamin \|  \|  \| \| Vitamins and vitamin metabolism \| niacinamide \|  \|  \| \| Vitamins and vitamin metabolism \| pantothenic acid \|  \|  \| \| Vitamins and vitamin metabolism \| pyridoxamine-phosphate \|  \|  \| \| Vitamins and vitamin metabolism \| riboflavine \|  \|  \| \| Vitamins and vitamin metabolism \| thiamine \|  \|  \| \| Vitamins and vitamin metabolism \| thiamin-monophosphate \|  \|  \| \| Vitamins and vitamin metabolism \| threonic acid \|  \|  \| \| Vitamins and vitamin metabolism \| X-2558-possible-N1-methyl-2-pyridone-5-carboxamide-and-others \|  \|  \| |
|  |
|  |
|  |
|  |
|  |
|  |
